# Exosomal miRNAs Mediate Immune–Metabolic Interactions in the Hemocytes of the Pearl Oyster *Pinctada fucata martensii*

**DOI:** 10.3390/ani15202955

**Published:** 2025-10-13

**Authors:** Ping Wang, Chaoxuan Wu, Yalin Xu, Minxin Liang, Wanqi Tan, Qingheng Wang, Yuewen Deng, Zhe Zheng

**Affiliations:** 1 Fishery College, Guangdong Ocean University, Zhanjiang 524088, China; 17616195967@163.com (P.W.); w2541349090@163.com (C.W.); 13662272418@stu.gdou.edu.cn (Y.X.); 2112201065@stu.edu.cn (M.L.); iukikisakura@163.com (W.T.); wangqingheng@163.com (Q.W.); dengyw@gdou.edu.cn (Y.D.); 2 Pearl Breeding and Processing Engineering Technology Research Centre of Guangdong Province, Zhanjiang 524088, China; 3 Guangdong Science and Innovation Center for Pearl Culture, Zhanjiang 524088, China; 4 Guangdong Provincial Key Laboratory of Aquatic Animal Disease Control and Healthy Culture, Zhanjiang 524088, China; 5 Guangdong Provincial Engineering Laboratory for Mariculture Organism Breeding, Zhanjiang 524088, China; 6 Guangdong Marine Ecology Early Warning and Monitoring Laboratory, Zhanjiang 524088, China

**Keywords:** exosomal microRNAs, immune–metabolic regulation, *Pinctada fucata martensii*, molluscan immunity

## Abstract

**Simple Summary:**

The development of marine pastures is vital for sustainable blue economies but faces increasing threats, such as disease outbreaks. This study investigates how pearl oysters, an economically important bivalve, utilize exosomes and their cargo microRNAs to regulate key immune and metabolic pathways—including apoptosis, inflammation, and metabolic reprogramming—following lipopolysaccharide (LPS) stimulation. Our findings elucidate the innate disease-resistance mechanisms in bivalves and suggest that exosomal miRNAs could serve as potential biomarkers for assessing their health in aquaculture settings.

**Abstract:**

Mollusks, such as bivalves, face increasing threats, such as disease, in aquaculture. Exosomes, widely derived from living cells carrying diverse bioactive molecules, affect the immune response. To overcome these challenges, bivalves utilize exosomal miRNAs as critical regulators of immune responses. This study investigates the role of exosomal miRNAs in modulating immune and metabolic responses in *Pinctada fucata martensii* following lipopolysaccharide (LPS) stimulation. Exosomes (75–150 nm) were isolated from hemolymph and characterized. High-throughput sequencing identified 30 differentially expressed miRNAs (DEMs) and 1349 differentially expressed genes (DEGs) in LPS-treated oysters, with significant enrichment in TNF, TLR/NF-κB, and metabolic pathways. This study revealed exosomal miRNA-mediated regulation of immune genes (IκBα, TRAF6, IRAK1, and BIRC2/3) and metabolic enzymes (PCK and CYP2J), demonstrating their role in apoptosis, inflammation, and metabolic reprogramming. Network analysis highlighted miRNA–mRNA interactions, including miR-7/IκBα (TNF pathway) and miR-34_5/IRAK1 (TLR pathway). Additionally, exosomal miRNAs (miR-92_2 and novel_mir5) were found to regulate oxidative stress (SOD1) and gluconeogenesis (PCK), linking immune defense with metabolic adaptation. These findings provide novel insights into exosomal miRNA-mediated immune regulation in bivalves, revealing conserved mechanisms with potential implications for molluscan health and disease management.

## 1. Introduction

Mollusks represent a crucial aquaculture group, accounting for approximately 27% of global aquaculture production, and serve as a vital source of high-quality protein for human consumption [1]. However, most molluscan aquaculture, including oysters and scallops, relies on open-pond farming. These systems face growing challenges due to climate change, population growth, coastal environmental degradation, and increasing pathogenic threats, posing significant challenges to shellfish farming and affecting the immune defense system of shellfish. Lipopolysaccharide (LPS), a classic pathogen-associated molecular pattern (PAMP), is widely used to study shellfish immunity due to its specific activation of defined signaling pathways (e.g., TLR/NF-κB) [2]. To ensure sustainable aquaculture and develop disease-resistant breeds, a deeper understanding of the immune regulatory mechanisms in shellfish is essential. Mollusks possess an open circulatory system in which hemocytes serve as the primary effectors of both cell-mediated and humoral immunity, actively encountering and neutralizing foreign substances. These phagocytic cells play a central role in immune defense: upon exposure to bacterial antigens, hemocytes migrate to sites of infection, adhere to pathogens, and engulf them via pseudopodia formation, ultimately digesting the invaders within phagosomes [3]. As invertebrates, mollusks primarily rely on their innate immune system to combat pathogens, employing evolutionarily conserved signaling pathways such as toll-like receptor (TLR) and tumor necrosis factor (TNF) pathways that share homology with vertebrate systems [4]. This signaling subsequently triggers the respiratory burst in hemocytes, generating reactive oxygen intermediates (ROIs), including superoxide anions (O_2_^−^), hydroxyl radicals (OH·), hydrogen peroxide (H_2_O_2_), and singlet oxygen (^1^O_2_), that directly eliminate microbes such as bacteria, fungi, and protozoa [5].

MicroRNAs (miRNAs) are endogenous, non-coding small RNAs (20~25 nucleotides) that play critical roles in gene regulation [6]. Since the discovery of the first miRNA, lin-4, in the nematode *Caenorhabditis elegans* in 1993 [7], advances in high-throughput sequencing have revealed both conserved and species-specific miRNAs across taxa [8]. Emerging evidence highlights their importance in immune regulation. For example, in *Penaeus monodon*, miR-145 and miR-454 regulate the expression of the PmTRIAPI gene, thereby controlling the apoptosis of hemocytes [9]. In *Pinctada fucata martensii*, miR-29a activates its immune response by downregulating the expression of Y2R [10]. Similarly, miR-4047 regulates the NF-kB signaling pathway via myeloid differentiation primary-response protein 88 (MyD88) gene suppression, and miR-516b-5p mediates the cholinergic anti-inflammatory signaling pathway by targeting the nicotinic acetylcholine receptor β subunit (nAChRβ) [11].

Exosomes (30–150 nm extracellular vesicles) are formed via multivesicular endosome (MVE) maturation and plasma membrane fusion, facilitating intercellular communication by transporting nucleic acids, lipids, proteins, and metabolites [12]. They are released into the extracellular space following the fusion of MVEs with the plasma membrane [13,14]. Exosomes play a crucial role in modulating immune responses, the immune microenvironment, and self-immune regulation [15]. Exosomal miRNAs play an important regulatory role in the body’s immune response. For instance, porcine milk exosomal miR-4334 and miR-219 inhibit cellular inflammation through the NF-κB pathway, while miR-338 prevents apoptosis through the p53 pathway [16]. After infection with *Vibrio parahaemolyticus*, exosomal miR-224 in *Scylla paramamosain* targets heat shock protein 70 (HSP70), activating tumor necrosis factor receptor-associated factor 6 (TRAF6), to modulate the production of mitochondrial reactive oxygen species (mROS) and anti-lipopolysaccharide factors (ALFs), ultimately defending against the invasion of pathogens [17]. Furthermore, immune-related miRNAs such as miR-2a/b/c and miR-92 have also been detected in the hemolymph exosomes of *P. f. martensii* after graft transplantation [12], highlighting their immunological significance. Therefore, studying the immune regulatory mechanisms of exosomal miRNA in *P. f. martensii* holds significant importance.

*P. f. martensii* serves as a critically important aquaculture species for pearl production in southern China’s marine ecosystems. However, the industry now faces mounting sustainability challenges due to escalating disease outbreaks and environmental degradation, which have led to significantly increased mortality rates. While exosomal miRNAs are well-established as key regulators of immune responses in marine bivalves through their modulation of hemocyte-mediated defenses, the primary immune mechanism in these organisms, their specific regulatory mechanisms in *P. f. martensii* remain unexplored, creating a critical knowledge gap for this economically vital species. To address this pressing issue, this study employs RNA sequencing (RNA-seq) to systematically investigate bacterial LPS-stimulated immune regulation by analyzing exosomal miRNA and hemocyte mRNA expression profiles. This study aimed to construct a comprehensive regulatory network. We hope the findings of this study provide fundamental insights into the molecular basis of pearl oyster immunity through characterization of miRNA–hemocyte interactions, and practical applications for developing disease early-warning systems and guiding selective breeding of stress-resistant varieties to enhance aquaculture sustainability.

## 2. Materials and Methods

### 2.1. Laboratory Animals

Healthy *P. f. martensii* (1.5 years old, shell length 5–6 cm, weight 25 ± 3 g) used in the experiment were provided by the Daqiuzhuang Aquatic Products Company farm located in the Liusha Bay area of Zhanjiang, China (109°57′ E, 20°25′ N). All individuals were collected from the same cultivation cage to ensure consistency in genetic background and rearing conditions as much as possible. Prior to experimentation, the pearl oysters were acclimated in a flow-through seawater system maintained at 25–27 °C for one week to mitigate transport-induced stress and ensure physiological stabilization. The seawater was rich in natural microalgae and nutrients, which adequately satisfied the basal energy requirements of the shellfish during the acclimation phase. Therefore, no artificial feeding was administered throughout the experimental period.

### 2.2. Immune Stimulation and Separation of Serum and Hemocytes

Ninety healthy *P. f. martensii* were randomly allocated into two treatment groups, each with three replicates, namely the experimental group (LPS_1, LPS_2, LPS_3) and the control group (PBS_1, PBS_2, PBS_3). The control group received an intramuscular injection of 100 μL PBS (0.5 mg/mL) into the adductor muscle, while the experimental group was injected with 100 μL LPS (0.5 mg/mL; Sigma-Aldrich, catalog number: L5293; Shanghai, China) via the same route. Following injection, all oysters were maintained under standard aquaculture conditions (temperature: 25–27 °C; salinity: 30–32 ppt; pH: 8.0–8.2; flow-through seawater system with a 2×/h turnover rate) for a 12 h experimental period. Hemolymph samples were subsequently collected using sterile techniques and centrifuged at 3500 rpm for 10 min at 4 °C to separate serum and hemocyte fractions, which were immediately flash-frozen in liquid nitrogen and stored at −80 °C until analysis. Each group had 15 oysters that were pooled together for sequencing using DNBSEQ.

### 2.3. Exosome Isolation and Characterization from Serum

Exosomes were isolated from *P. f. martensii* serum using differential centrifugation and ultrafiltration. Briefly, the serum was first centrifuged at 4 °C and 4450 rpm for 10 min to remove cellular debris, followed by a second centrifugation at 5600 rpm for 15 min to further clarify the supernatant. The resulting supernatant was filtered through a 0.22 μm membrane (Yesen, cat. no. 84301ES03) and concentrated using a 30 kDa molecular-weight cutoff ultrafiltration tube. Exosomes were then precipitated using the ExoQuick™ Exosome Precipitation Solution Kit (System Biosciences, Palo Alto, CA, USA).

For characterization, exosome size distribution and concentration were determined via nanoflow cytometry (NanoFCM, Flow NanoAnalyzer U30E; Shanghai, China). Morphological analysis was performed by negative-staining transmission electron microscopy (TEM). A 20 μL aliquot of the purified exosome suspension was applied to a copper grid, fixed with 2% uranyl acetate for 5 min, and visualized using a Zeiss Libra 120 TEM (Shanghai, China) at 80 kV.

### 2.4. Library Construction and Sequencing of Exosomal miRNA and Hemocyte mRNA

The microRNAs from serum exosomes were extracted using the TransExoTM Serum/Plasma Exosome miRNA Extraction Kit (Shanghai, China). The integrity and concentration of the samples were assessed using the Agilent 2100 Bioanalyzer (Agilent RNA 6000 Nano Kit; Shanghai, China). A small RNA library was constructed using the VAHTS Small RNA Library Prep Kit for Illumina. One microgram of RNA samples was used, and 18–30 nt small RNAs were recovered by 15% denaturing polyacrylamide gel (containing 7–8 M urea; Baiaonolai, cat. no. YTB0218) electrophoresis. The obtained small RNAs were ligated with 5′ and 3′ adapters, and reverse transcription extension was performed with a UMI-containing RT primer to synthesize single-stranded cDNA. Reaction conditions: 42 °C for 1 h; 70 °C for 15 min. PCR amplification conditions: 95 °C for 3 min; 98 °C for 20 s, 56 °C for 15 s, 72 °C for 15 s, for a total of 20 cycles; 72 °C for 10 min. PCR products in the 110–130 bp range were separated by PAGE electrophoresis, and the separated products were sent to the sequencing company for sequencing. Sequencing was performed on the BGISEQ-500 sequencer (Shanghai, China), and various types of small RNAs, including miRNAs, were identified using the AASRA.20 analysis software.

Total RNA from hemocytes was extracted using the Trizol method. The integrity of the total RNA was assessed by 1% agarose gel electrophoresis, and the concentration and purity of the total RNA were determined using a micro-ultraviolet nucleic acid-protein quantifier. mRNA was purified from the total RNA using either mRNA enrichment or rRNA removal methods. Fragmented mRNA was subjected to buffer fragmentation, followed by reverse transcription with random N6 primers to synthesize double-stranded cDNA. The 5′ ends were phosphorylated, and bubble-like adapters were formed at the 3′ ends. PCR amplification with specific primers and circularization of single-stranded DNA with bridge primers resulted in a single-stranded circular DNA library, which was then subjected to DNBSEQ sequencing.

### 2.5. Screening of Differentially Expressed Exosomal miRNAs and Prediction of Target Genes

The *p*-value of the differential test was subject to multiple test correction, and the threshold for the *p*-value was determined by controlling the False Discovery Rate (FDR). An FDR of less than 0.05 indicates significant differences in the results. Referring to the *P*. *f*. *martensii* genome sequence [18], the CDS sequences and 3′ UTR sequences of the genes were obtained. The target genes of the differentially expressed miRNAs were predicted using RNAhybrid (http://bibiserv.cebitec.uni-bielefeld.de/rnahyb; (accessed on 15 August 2024)) and miRanda (http://www.microrna.org/ (accessed on 15 August 2024)). The target genes were functionally annotated with reference to the NR, KEGG, and InterPro databases. Significantly enriched pathways from the KEGG database were obtained with a threshold of Q-value ≤ 0.05. Based on the crosstalk relationships between pathways, the regulatory network of exosomal miRNAs and genes in the signaling pathways was drawn using the BioRender software (https://www.biorender.com/; accessed on 1 January 2025).

### 2.6. Analysis of Differentially Expressed mRNA in Hemocytes

The raw image data obtained from sequencing were transformed into raw reads through base calling and filtered using SOAPnuke (version 1.4.0) to ultimately obtain clean reads. Bowtie2 (version 2.2.5) was used to map the clean reads of each sample to the reference sequence after the splicing of new transcripts, and RSEM (version 1.2.8) was used to calculate the gene expression levels of each sample. The Pearson correlation coefficients between each pair of samples were calculated using the cor and princomp functions in R software (version 4.5.0).

Differentially expressed gene (DEG) analysis was performed between the PBS and LPS groups using DESeq2, with the screening criteria set to |log_2_Fold Change| > 1 and Q-value ≤ 0.05. GO enrichment analysis and KEGG enrichment analysis were performed using the phyper function in R software, considering Q-values < 0.05 and Q-values < 0.055 as significantly enriched.

### 2.7. Analysis of the Targeting Relationship Between Differentially Expressed Exosomal miRNAs and Differentially Expressed mRNAs in Hemocytes

Using miRanda (version 3.3a) with a threshold of score = 140 and energy = −20, the target genes of differentially expressed miRNAs were predicted from both the 3′UTR and CDS regions. The predicted target genes were intersected with the differentially expressed mRNAs annotated to the KEGG pathways. By analyzing the expression levels of differentially expressed genes (DEGs) and differentially expressed miRNAs (DEMs), an immune-related miRNA–mRNA regulatory network was constructed. Visualization of this network was performed using Cytoscape (version 3.10.0) and BioRender drawing software (https://www.biorender.com/; accessed on 22 January 2025).

### 2.8. qRT-PCR Validation of miRNA-Seq and mRNA-Seq

To verify the accuracy of the miRNA-seq and mRNA-seq data, qRT-PCR experiments were conducted on 3 randomly selected miRNAs and 3 genes. U6 was used as the internal reference for miRNA, and GAPDH as the internal reference gene. Due to the low concentration of microRNA in exosomes, we performed in vitro transcription according to the instructions of the TransScript miRNA First-Strand cDNA Synthesis SuperMix (TRAN, AT351; Shanghai, China) kit, using the maximum recommended volume of 20 μL. Following the protocol of the miRcute Plus miRNA qPCR Kit (SYBR Green, FP411; Shanghai, China), 0.4 μL of the in vitro transcribed cDNA was added to a total reaction system of 10 μL for detection. The primers were diluted 10-fold before use. Primers were designed based on the miRNA-seq data and the *P. f. martensii* database using Primer Premier 5.0 (Table 1).

An amount of 8 µL of miRNA was reverse-transcribed using the miRcute Enhanced miRNA cDNA First Strand Synthesis Kit to obtain the required cDNA template. miRNA-specific primers were used, and the reaction system was configured according to the instructions of the miRcute Enhanced miRNA Fluorescence Quantitative Detection Kit (Shanghai, China) and detected using the LightCycler^®^ 96 Real-Time Fluorescence Quantitative PCR instrument (Shanghai, China). The qRT-PCR reaction program included 95 °C for 15 min; 94 °C for 20 s, 60 °C for 34 s, for a total of 45 cycles; melting curve analysis for 1 cycle: 95 °C for 10 s, 65 °C for 60 s, 97 °C for 1 s; 37 °C for 30 s.

cDNA was synthesized using the HiScript III All-in-one RT SuperMix Perfect (Shanghai, China) for qPCR. The qRT-PCR reaction system consisted of 0.4 µL each of forward and reverse primers, 3.8 µL RNase-free ddH_2_O, 0.4 µL cDNA, and 5 µL ChamQ Universal SYBR qPCR Master Mix. Detected using the LightCycler^®^ 96 Real-Time Fluorescence Quantitative PCR instrument, the qRT-PCR reaction program included 95 °C for 300 s; 95 °C for 10 s, 60 °C for 15 s, 72 °C for 15 s, for a total of 40 cycles; 95 °C for 10 s, 60 °C for 60 s, 95 °C for 1 s; 37 °C for 30 s.

The qRT-PCR data were processed using the 2^−ΔΔCt^ method, and the calculated results were analyzed for significant expression levels using SPSS 24.0. A *p*-value of less than 0.05 indicates a statistically significant difference.

## 3. Results

### 3.1. Isolation and Identification of Exosomes

**Figure 1:**
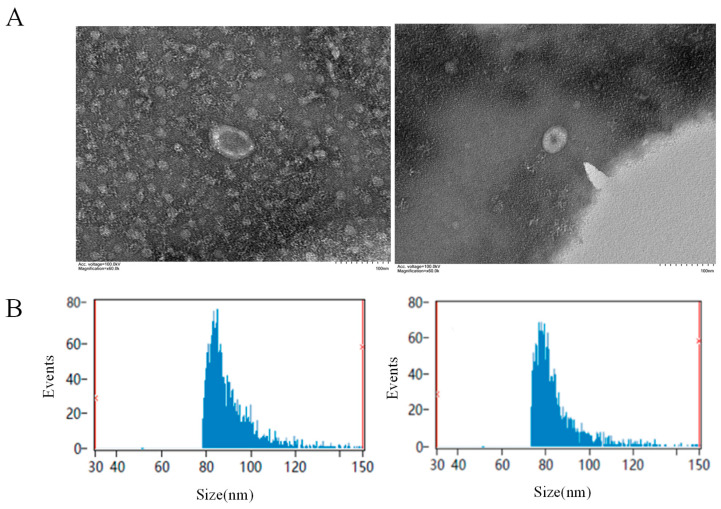
Identification of hemolymph exosomes. (**A**) Results of exosome electron microscopy. The left panel shows the PBS group, and the right panel shows the LPS group. (**B**) Analysis of exosome particle size. The left panel shows the PBS group, and the right panel shows the LPS group.

Exosomes from hemolymph of the oysters in the PBS and LPS groups were observed and analyzed for particle size using a transmission electron microscope. The results showed that the exosomes appeared as round or oval vesicles, with a visible membrane structure around the vesicles, which was intact and had a clear outline (Figure 1A). The size range was 75–150 nm, and the average size of the exosomes from the PBS and LPS groups was 90.8 nm and 89.41 nm, respectively (Figure 1B).

### 3.2. Analysis of Exosomal miRNA-seq

**Figure 2:**
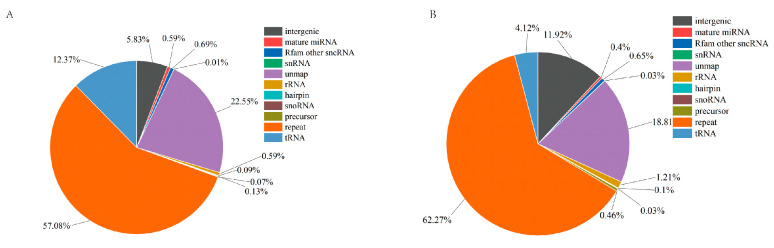
Identification of small RNAs in exosomes from the PBS and LPS groups. (**A**) PBS group, (**B**) LPS group.

High-throughput sequencing of hemolymph exosomal miRNAs in *P. f. martensii* using BGISEQ-500 technology generated 27,247,000 and 26,317,926 high-quality clean tags from the PBS and LPS groups, respectively, with genome alignment rates of 75.26% and 79.77%. Small RNA annotation revealed that miRNAs represented 0.80% and 1.00% in PBS and LPS groups, respectively, of total small RNAs (Figure 2), identifying 68 mature miRNAs (85 precursors) in the PBS group and 71 mature miRNAs (87 precursors) in the LPS group, while miRDeep2 analysis predicted 18 novel miRNAs common to both groups.

**Figure 3:**
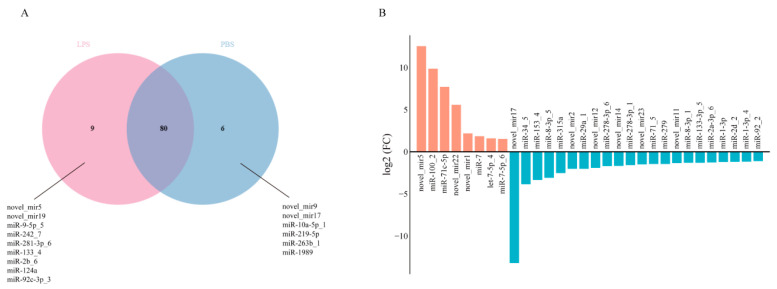
miRNA differential expression profiles. (**A**) Venn diagram of miRNA expression in the PBS and LPS groups. The red section represents miRNAs expressed solely in the LPS group, the blue section represents miRNAs expressed solely in the PBS group, and the overlapping section indicates genes commonly expressed in both groups. (**B**) Bar chart of differentially expressed miRNAs between the PBS and LPS groups. The red section indicates upregulated miRNAs, and the blue section indicates downregulated miRNAs.

Comparative miRNA expression analysis revealed both shared and distinct profiles between experimental groups: 80 miRNAs were co-expressed in the PBS and LPS groups, while 6 and 9 miRNAs showed exclusive expression in the PBS and LPS groups, respectively (Figure 3A). Differential expression analysis (|log_2_FC| > 1, *p* ≤ 0.05) identified 30 significant DEMs, comprising 8 upregulated and 22 downregulated miRNAs in LPS-treated oysters compared to PBS-treated groups (Figure 3B, Table S1).

### 3.3. Hemocyte mRNA-seq Analysis

**Figure 4:**
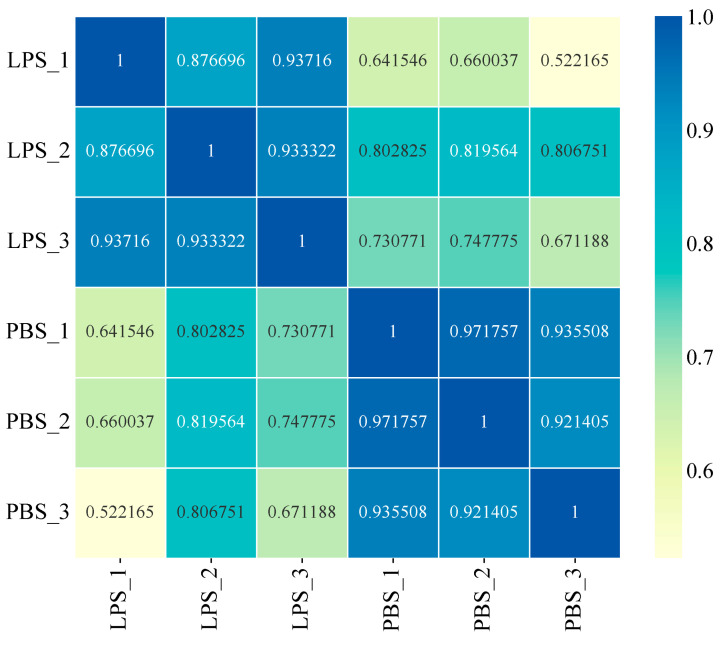
Heatmap of correlation between six samples. Note: The vertical and horizontal axes represent the sample names, while the color variation indicates the square of the Pearson correlation coefficient (R^2^) between samples.

The sequencing data demonstrated high quality across all samples, with clean read ratios ≥ 96.24%, Q20 scores ≥ 97.19%, and Q30 scores ≥ 92.69% (Table 2), meeting stringent standards for transcriptomic analysis. Genome alignment rates ranged from 61.33% to 66.80% for the *P. f. martensii* reference genome, while high intra-group reproducibility was confirmed by Pearson correlation coefficients (R^2^ > 0.87) between biological replicates (Figure 4). These quality metrics collectively validate the reliability of our mRNA-seq dataset for downstream differential expression analysis.

**Figure 5:**
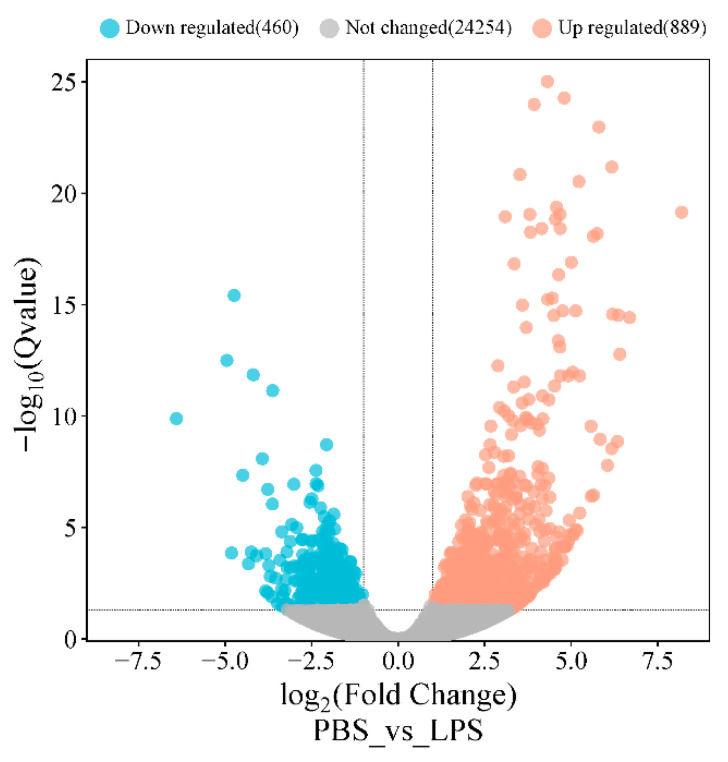
Volcano plot of mRNA expression in the PBS and LPS groups. Note: Each dot represents a gene, with red dots indicating upregulated genes (log_2_Fold Change > 1, Q-value ≤ 0.05), blue dots indicating downregulated genes (log_2_Fold Change < −1, Q-value ≤ 0.05), and gray dots representing genes with no significant differential expression.

**Figure 6:**
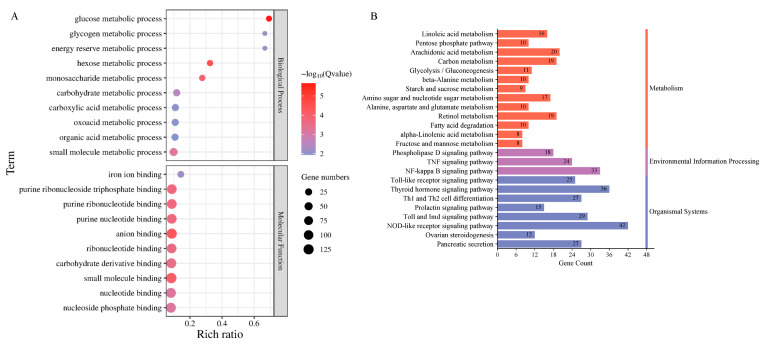
Differentially expressed mRNA enrichment analysis. (**A**) GO enrichment analysis of differentially expressed mRNAs, showing the top ten GO terms significantly enriched (Q-value < 0.05) among differentially expressed mRNAs. The vertical axis represents the GO terms, while the Rich ratio indicates the ratio of the number of differentially expressed genes in a particular GO term to the total number of genes. The size and color of the dots represent the number of differentially expressed genes in that GO term and the −log_10_(Q-value) value, respectively. A redder color indicates a smaller Q-value and a more significant enrichment. (**B**) KEGG enrichment analysis of differentially expressed mRNAs. The KEGG pathways significantly enriched (Q-value < 0.055) by differentially expressed mRNAs are also shown. The vertical axis represents the KEGG pathways, while the horizontal axis indicates the number of differentially expressed genes in each KEGG pathway. Different colors represent different top-level categories.

Transcriptomic analysis identified 1349 differentially expressed genes (DEGs) (|log_2_FC| > 1, Q-value ≤ 0.05) between groups, comprising 889 upregulated and 460 downregulated genes (Figure 5, Table S3). Functional annotation revealed 705 DEGs mapped to 1802 GO terms, with significant enrichment (Q-value < 0.05) in metabolic processes (33 BP terms, including glucose and carbohydrate metabolism) and molecular functions (35 MF terms, particularly anion and small-molecule binding) (Figure 6A). KEGG analysis of 652 DEGs identified 241 pathways, with 24 significantly enriched (Q-value < 0.055) in key immune–metabolic processes, including glycolysis/gluconeogenesis, TNF signaling, and NF-κB signaling pathways (Figure 6B).

### 3.4. Exosomal DEMs Regulate Immune-Related DEGs

**Figure 7:**
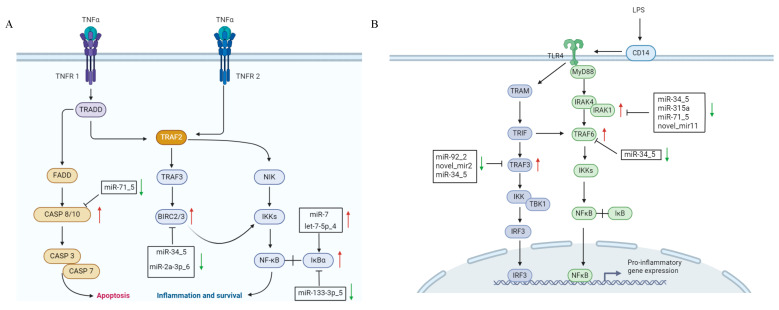
The regulatory role of exosomal miRNAs in immune-related signaling pathways of hemocytes. (**A**) TNF signaling pathway. (**B**) TLR signaling pathway. Red arrows indicate upregulated expression, while green arrows indicate downregulated expression.

**Figure 8:**
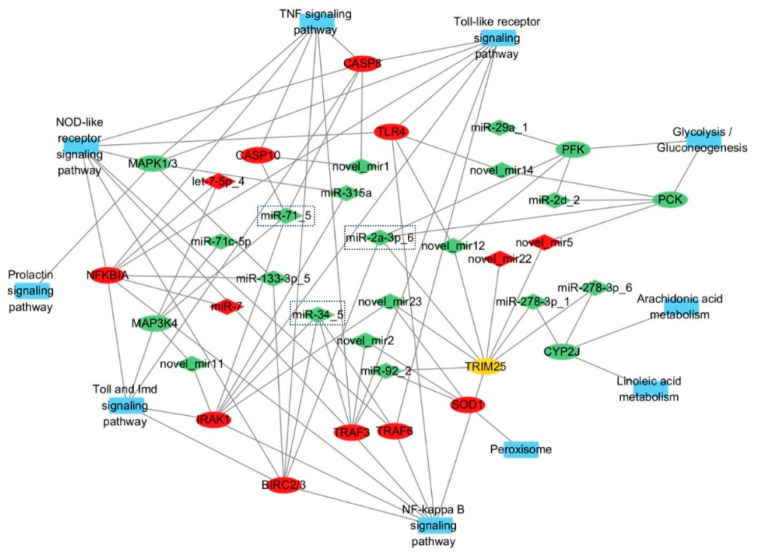
Immune-related miRNA–mRNA regulatory network. Note: Rectangles represent the KEGG pathways where differentially expressed genes are located, ellipses represent differentially expressed genes, and diamonds represent differentially expressed miRNAs. Red indicates upregulated expression, green indicates downregulated expression, and yellow indicates that multiple genes encode the same protein, with both upregulated and downregulated genes present.

Using miRanda (v3.3a), we predicted 13,199 target genes for the 30 differentially expressed miRNAs (30 DEMs), comprising 12,211 CDS and 2254 3′UTR targets, including 684 DEGs post-LPS stimulation (Table S2). Functional enrichment showed that DEMs modulate key immune pathways: (1) In the TNF signaling pathway, miR-7/let-7-5p_4/miR-133-3p_5 target IκBα, miR-34_5/miR-2a-3p_6 target and negatively regulate BIRC2/3, and miR-71_5 targets and negatively regulates CASP8/10, collectively regulating hemocyte apoptosis; IκBα, BIRC2/3, and CASP8/10 regulate inflammation and apoptosis in the TNF signaling pathway. IκBα controls the initiation of inflammation by inhibiting NF-κB activity; BIRC2/3 maintains cell survival by suppressing apoptotic signals; and CASP8/10 induce apoptosis by activating the caspase cascade. Dysfunction of these molecules may lead to abnormal apoptosis or excessive inflammation, thereby affecting the pathogenesis of various diseases [19]. (2) In TLR signaling, miR-92_2/novel_mir2 target TRAF3, while miR-34_5/miR-315a/miR-71_5/novel_mir11 target IRAK1, with miR-34_5 additionally regulating TRAF6/TRAF3 to modulate inflammation; TRAF3 balances immune responses by modulating the production of type I interferons and inflammatory cytokines. IRAK1, acting as a kinase, activates downstream NF-κB and MAPK signaling pathways to promote inflammation. TRAF6 regulates inflammation and cell survival by activating NF-κB and MAPK pathways. The coordinated actions of these molecules are crucial for maintaining immune homeostasis and combating pathogen infections [20]. (3) In peroxisome pathways, miR-92_2/novel_mir2/novel_mir23 target SOD1 to mitigate ROS-induced oxidative stress. Metabolic regulation was also observed, with novel_mir5 inhibiting gluconeogenesis via PCK targeting and miR-278-3p_6/1 regulating lipid metabolism through CYP2J-mediated arachidonic/linoleic acid pathways (Figure 7). Network analysis (Figure 8) revealed complex miRNA–mRNA interactions, demonstrating both miRNA pleiotropy (single miRNAs regulating multiple mRNAs) and combinatorial regulation (multiple miRNAs co-targeting single mRNAs). The results indicate that miR-71_5, miR-34_5, and miR-2a-3p_6 have the highest number of target genes and the strongest regulatory capabilities.

### 3.5. qRT-PCR Verification of miRNA-seq and mRNA-seq

**Figure 9:**
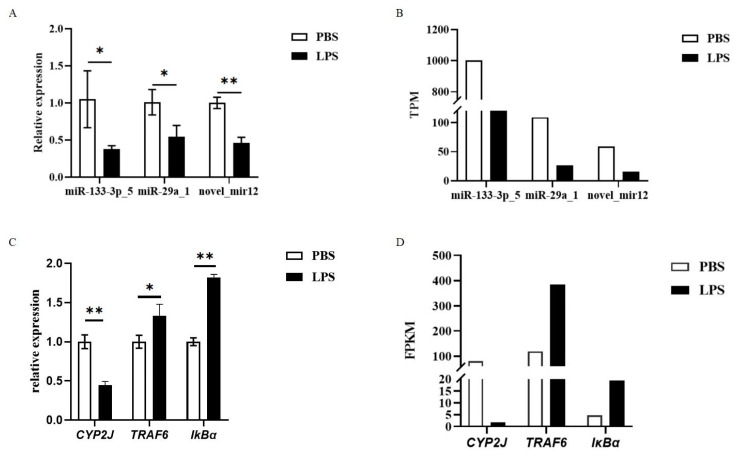
Results of qRT-PCR validation for miRNA-seq and mRNA-seq. (**A**) Differential expression of three miRNAs validated by qRT-PCR. (**B**) Differential expression of three miRNAs obtained from miRNA-seq. (**C**) Differential expression of three genes validated by qRT-PCR. (**D**) Differential expression of three genes obtained from mRNA-seq. * indicates significant difference; ** indicates extremely significant difference.

Using U6 and GAPDH as reference genes for miRNA and mRNA normalization, respectively, qRT-PCR analysis (2^−ΔΔCt^ method) confirmed the sequencing data: exosomal miR-133-3p_5, miR-29a_1, and novel_mir12 were significantly downregulated (*p* < 0.05) in LPS-stimulated hemolymph (Figure 9A,B), aligning with miRNA-seq results. Hemocyte mRNA analysis revealed significant downregulation of CYP2J and upregulation of TRAF6 and IκBα (*p* < 0.05) (Figure 9C,D), consistent with mRNA-seq findings. Statistical significance was validated using SPSS 24.0.

## 4. Discussion

In this study, we successfully isolated exosomes from the hemolymph of LPS-stimulated *P. f. martensii*, with transmission electron microscopy and nanoparticle tracking analysis confirming their characteristics and size range as consistent with exosomes from higher vertebrates [21]. Transcriptomic analysis at 12 h post-LPS stimulation identified DEMs and DEGs linked to the immune-related pathways, including lipid metabolism (linoleic acid metabolism), energy metabolism (glycolysis/gluconeogenesis), and immune signaling cascades (TNF, NF-κB, TLR, and NLR pathways) upon KEGG pathway analysis.

After LPS stimulation, Lu also detected a significant enrichment of NF-κB [22]. Following LPS stimulation, scallops exhibit a typical immune activation response, characterized by the upregulation of key genes in the TLR and NF-κB signaling pathways [23]. The activation of these pathways enables host cells to rapidly recognize pathogens and enhances the intensity and duration of the immune response.

Importantly, we identified multiple miRNA–mRNA regulatory axes and found that miRNAs exert strong regulatory effects, such as miR-7/IκBα in TNF signaling, miR-34_5/IRAK1 in TLR signaling, that collectively modulate the oyster’s immune response to bacterial challenge. The TNF signaling pathway plays a pivotal role in apoptosis by regulating cell survival decisions during bacterial challenge [24]. Wu et al. [25] demonstrated TNFR expression in *P. f. martensii* hemocytes, showing upregulation of both PmTNFR1 and PmTNFR2 following LPS stimulation. Furthermore, PmTNFR1 silencing suppressed downstream effectors, including TRADD, Caspase-8/3, TRAF2, NIK, IKK, and NF-κB, confirming the existence of a functional TNFR1-mediated apoptosis pathway in *P. f. martensii* [25]. Our current findings align with these observations, revealing LPS-induced upregulation of multiple TNF pathway components (Caspase-3/7/8/10, NF-κB, IκBα, BIRC2/3, and TRAF3). This suggests regulatory mechanisms that promote apoptosis to eliminate infected hemocytes and prevent excessive cell death that could disrupt immune homeostasis. This delicate balance is vital for the TNF-mediated immunity in bivalves. In the TLR signaling pathway, we identified two key regulatory networks, notably downregulated miR-92_2 and novel_mir2, which target the TRAF3 3′UTR, correlating with TRAF3 upregulation, suggesting their role as negative regulators of inflammatory signaling [26]. Additionally, a cluster of downregulated miRNAs (miR-34_5, miR-315a, miR-71_5, and novel_mir11) that interact with the IRAK1 3′UTR, with concomitant IRAK1 upregulation, were identified. miR-34_5 also targets the CDS regions of both TRAF6 and TRAF3, demonstrating its multi-faceted regulatory capacity. These coordinated miRNA–mRNA interactions create a synergistic pro-inflammatory effect by releasing inhibition on critical signaling nodes (TRAF3, IRAK1, TRAF6), thereby amplifying TLR4 signal transduction, enhancing NF-κB activation, and potentiating inflammatory mediator production [27]. The findings further establish exosomal miRNAs as among the regulators of inflammatory homeostasis in pearl oyster immunity through their integrated control of multiple pathway components.

When confronting pathogen infection, *P. f. martensii* employs dual strategies of resistance (pathogen clearance) and tolerance (damage control), where LPS stimulation rapidly activates TLR-mediated inflammatory responses in hemocytes within 12 h. The inflammatory response generates bactericidal reactive oxygen species (ROS) to kill or inhibit bacterial growth [28]. In *C. gigas*, *M. galloprovincialis*, and *A. irradians*, ROS is produced during phagocytosis; however, excessive ROS causes collateral damage through lipid peroxidation and DNA breaks [5]. To mitigate this, pearl oysters upregulate antioxidant enzymes like superoxide dismutase (SOD), catalase (CAT), and peroxidase (PO) to convert ROS such as superoxides (O^2-^) and hydrogen peroxide (H_2_O_2_) into less harmful molecules such as oxygen and water [29]. Studies have reported elevated PmSOD1 expression and increased SOD activity post-LPS challenge, consistent with findings in scallops, *C. farreri*, and *P. f. martensii* [23,30]. Crucially, our study reveals a novel regulatory mechanism: three exosomal miRNAs (miR-92_2, novel_mir2, novel_mir23) targeting the PmSOD1 CDS region are significantly downregulated after LPS stimulation, potentially releasing their suppression of PmSOD1 to bolster antioxidant defenses. This coordinated response, involving both TLR pathway activation and miRNA-mediated SOD regulation, demonstrates how *P. f. martensii* maintains oxidative balance during immune challenges, mirroring protective miR-92a effects observed in human endothelial cells [31] while showcasing unique molluscan adaptations.

We found that some miRNAs possess strong regulatory capabilities and can modulate the expression of multiple target genes. For instance, mir-133-3p-5 can regulate the expression of NF-κB, BIRC2/3, and MAPK. NF-κB is a key transcription factor in the inflammatory response and can regulate the expression of various inflammatory factors, such as IL-1β, IL-6, and TNF-α [32]. BIRC2/3 are anti-apoptotic proteins, and their increased expression can inhibit apoptosis [33]. The MAPK pathway can regulate energy metabolism and influence cell proliferation and differentiation. It plays a crucial role in multiple biological processes, including inflammation, apoptosis, immune response, cell proliferation and differentiation, as well as metabolic regulation [34]. Three exosomal miRNAs were identified as key regulators of IκBα [35]. Particularly, upregulated miR-7 and let-7-5p_4, which bind the IκBα 3′UTR and potentially enhance its expression, and downregulated miR-133-3p_5, which may suppress IκBα. This coordinated regulation likely fine-tunes apoptosis by controlling NF-κB activity. Similarly, miR-34_5 targets the BIRC2/3 gene via the 3′UTR region, and miR-2a-3p_6 interacts with the CDS region of the BIRC2/3 gene, potentially activating NF-κB to inhibit apoptosis [36]. Conversely, downregulated miR-71_5, which interacts with the CASP8/10 CDS, may promote apoptosis by inducing the expression of these caspase genes [37]. Thus, these findings demonstrate the exosomal miRNA network that orchestrates hemocyte apoptosis through balanced regulation of pro- and anti-apoptotic factors in the TNF pathway.

Some studies have demonstrated that LPS stimulation upregulates both TRAF6 and NF-κB in *P. f. martensii* hemocytes [38,39]. Activated NF-κB induces significant expression of IκBα, which contains nuclear localization and export signals (NLS and NES, respectively) that facilitate nucleocytoplasmic shuttling. This mechanism retrieves nuclear NF-κB to the cytoplasm, preventing excessive NF-κB activation [40], a critical function since NF-κB overactivation can promote apoptosis [16]. Supporting this, Zhang et al. observed 5.28-fold upregulation of IκBα in *P. f. martensii* following *Vibrio alginolyticus* challenge, consistent with our mRNA-seq findings [41]. While these studies confirm IκBα’s role in apoptosis regulation, its additional immune functions in bivalves warrant further investigation. Comparative studies reveal evolutionary conservation of anti-apoptotic mechanisms, with human inhibitors of apoptosis (IAPs) such as c-IAP1/2 and XIAP [42], and expanded IAP gene families in mollusks like *C. gigas* [43]. In *C. farreri*, LPS upregulates CfIAP-1/2, potentially suppressing Caspase-3 [44], while *P. f. martensii* shows transient induction of BIRC2/3/4/7 post-*V. alginolyticus* infection [45]. Our observation of LPS-induced BIRC2/3 upregulation further supports IAP-mediated apoptosis regulation in pearl oysters, highlighting conserved yet species-specific control of this critical immune response.

Emerging reports suggest that multiple miRNAs play crucial roles in apoptosis regulation across diverse species. Studies in mammalian systems reveal that curcumin upregulates miR-7a/b to suppress hypoxia-induced apoptosis in mouse cardiomyocytes [46], while let-7 downregulation promotes apoptosis in mouse skeletal muscle cells [47]. Similarly, miR-133-3p overexpression induces apoptosis in thyroid cancer SW579 cells [48]. On the other hand, LPS-stimulated female mice downregulated miR-34a to reduce apoptosis through XIST gene modulation [49]. These regulatory mechanisms appear evolutionarily conserved in invertebrates, where the miR-2 family demonstrates apoptotic regulatory functions [8] and miR-71 contributes to immune responses [37] via the activation of hemocyte apoptosis in some species such as *Marsupenaeus japonicus* [50]. Collectively, these findings establish that miR-7, let-7, miR-133-3p, miR-34, miR-2, and miR-71 constitute a conserved network of apoptosis-regulating miRNAs operating across vertebrate and invertebrate species. Under normal conditions, these miRNAs act to suppress immune responses, thereby preventing excessive immune activation that could deplete energy reserves and disrupt the immune microenvironment. However, upon LPS stimulation, the expression levels of these miRNAs decrease, leading to the activation of genes involved in immune and metabolic regulation within immune pathways. Given their ability to regulate multiple target genes, these miRNAs can serve as molecular markers for identifying the immune status of individuals.

Comparative genomic studies demonstrate remarkable conservation of TLR4-mediated inflammatory pathways across molluscan species. In *C. gigas*, *Vibrio anguillarum* infection upregulates CgToll-1 in hemocytes [51], while *Haliotis discus hannai* exhibits LPS-induced overexpression of HdTLR, HdMyD88-2/X, HdIκB, and HdRel [52], leading to NF-κB activation and subsequent production of inflammatory mediators (NO, IL1β, TNFα, IL6, iNOS, COX2) [53]. Similarly, *Chlamys farreri* shows upregulated CfTLR, CfMyD88, CfTRAF6, and CfIKK1 post-LPS challenge [54,55]. In *P. f. martensii*, key components of this pathway, including PmTLR4 (peaking at 6 h post-LPS) [56], PmIKK [57], and PmTRAF3 (highly expressed in hemocytes) [58], have been identified. Our mRNA-seq data corroborate these findings, revealing LPS-induced upregulation of PmLBP, PmMyD88, PmIRAK1/4, PmTRAF3/6, PmNF-κB, and PmIκBα in pearl oyster hemocytes. These collective results confirm that mollusks possess a functional TLR4–NF-κB signaling axis capable of orchestrating inflammatory responses to bacterial pathogens, a crucial mechanism of innate immunity in bivalves.

## 5. Conclusions

In this study, we successfully demonstrated that LPS stimulation triggers an immune response in *P. f. martensii*, mediated by exosomal miRNAs that regulate key immune and metabolic pathways in hemocytes. We successfully identified exosomes carrying miRNAs that modulate apoptosis (via TNF signaling), inflammation (via TLR/NF-κB), oxidative stress (via SOD1), and metabolic adaptation (via PCK and CYP2J). Additionally, miRNA-mediated regulation of IκBα, BIRC2/3, TRAF3/6, and IRAK1 is vital for balancing pro- and anti-apoptotic signals. Metabolic shifts can also be driven by miRNA suppression of gluconeogenesis (PCK) and lipid metabolism (CYP2J). Therefore, the results of this study highlight the critical role of exosomal miRNAs in orchestrating immune–metabolic interactions, enhancing pathogen resistance while maintaining cellular homeostasis. Our findings provide new insights into molluscan immunity, revealing vital regulatory mechanisms with similarities to vertebrates and identifying potential biomarkers for oyster health assessment.

## Figures and Tables

**Table 1:** Primers used in qRT-PCR.

Primer	Sequence (5′→3′)
miR-29a_1	TAGCACCATTTGAAATCAGTTT
novel_mir12	TATCACAGCAGTCTTTGATGGCC
miR-133-3p_5	TTGGTCCCCTTCAACCAGCTGT
U6-R	ATTTGCGTGTCATCCTTGC
U6-F	ATTGGAACGATACAGAGAAGATT
CYP2J-F	CCACCAGGACCCAGAGGAGT
CYP2J-R	CGCCATATTTCGCCCGTAG
TRAF6-F	GATGGAAACGCTTGTAGCGA
TRAF6-R	AGCACAGTCAAAGGGAGGAA
IκBα-F	AAATCGCAAGGTAAACGC
IκBα-R	AGTGACGGGTGGGAGCAT
GAPDH-F	GCAGATGGTGCCGAGTATGT
GAPDH-R	CGTTGATTATCTTGGCGAGTG

**Table 2:** Quality control results of mRNA-seq data.

Sample	LPS_1	LPS_2	LPS_3	PBS_1	PBS_2	PBS_3
Total raw reads (M)	43.82	43.82	43.82	43.82	43.82	43.82
Total clean reads (M)	42.34	42.49	42.17	42.43	42.63	42.76
Clean read ratio (%)	96.62	96.97	96.24	96.83	97.27	97.58
Total clean base (Gb)	6.35	6.37	6.33	6.36	6.39	6.41
Q20 (%)	97.28	97.4	97.22	97.22	97.19	97.3
Q30 (%)	93.00	93.23	92.86	92.76	92.69	92.97
Total mapping genome ratio (%)	63.22	64.09	62.87	62.95	61.33	66.80

## Data Availability

The original data used in this study are aggregated in the original text. If you need further consultation, please contact the corresponding author.

## Supplementary Materials

The following supporting information can be downloaded at: https://www.mdpi.com/article/10.3390/ani15202955/s1, Supplementary Table S1: 30 differentially expressed miRNAs in PBS-vs-LPS. Supplementary Table S2: Immune-related miRNA-mRNA target relationships. Supplementary Table S3: Expression of target genes interacting with miRNAs.
