# Supplementary material for: Exosomal miRNAs Mediate Immune–Metabolic Interactions in the Hemocytes of the Pearl Oyster Pinctada fucata martensii
Source: Animals (Basel). 2025 Oct 13;15(20):2955. doi: 10.3390/ani15202955 (PMC12560909; doi:10.3390/ani15202955)
Supplement: Supplementary file 1 [file animals-15-02955-s001.zip › animals-3832768-supplementary.pdf]

Supplementary table. 1 30 differentially expressed miRNAs in PBS-vs-LPS

miRNA id	TPM(PBS）	TPM(LPS)	log_2 Fold Change	Up/Down	P-value
novel_mir5	0.001	5.900456105	12.52661076	Up	0.0324454
miR-100_2	0.339512892	315.0243514	9.857781279	Up	1.08E-94
miR-71c-5p	0.339512892	70.50545007	7.698124613	Up	1.32E-21
novel_mir22	1.442929793	68.70531092	5.573348613	Up	1.84E-19
novel_mir1	167.6344906	752.1581418	2.165717009	Up	7.09E-88
miR-7	2.546346693	9.100703484	1.83754921	Up	0.0406418
let-7-5p_4	27.75517895	83.20643186	1.583938058	Up	7.84E-08
miR-7-5p_6	29.53762164	83.90648597	1.50622867	Up	3.12E-07
novel_mir17	9.506360986	0.001	-13.21467747	Down	0.00183394
miR-34_5	10.10050855	0.700054114	-3.850817676	Down	0.000911214
miR-153_4	15.27808016	1.500115959	-3.348317335	Down	0.000249722
miR-8-3p_5	8.487822309	1.000077306	-3.085282929	Down	0.020405
miR-315a	49.9932734	8.600664831	-2.539213904	Down	1.20E-08
novel_mir2	97.18556544	23.90184761	-2.02361991	Down	2.01E-12
miR-29a_1	109.1533949	26.90207953	-2.020567399	Down	1.00E-13
novel_mir12	59.07524327	15.7012137	-1.911677581	Down	1.19E-07
miR-278-3p_6	26.8215185	8.30064164	-1.692096151	Down	0.001658742
novel_mir14	19.77662598	6.200479297	-1.673344669	Down	0.00852832
miR-278-3p_1	218.1370333	73.2056588	-1.57520764	Down	1.64E-18
novel_mir23	55.68011435	19.90153839	-1.484282219	Down	1.77E-05
miR-71_5	254.5497911	93.50722811	-1.444798087	Down	5.08E-19
miR-279	525.3113227	194.0149974	-1.437004504	Down	3.77E-37
novel_mir11	14.68393259	5.800448375	-1.340002067	Down	0.0386828
miR-8-3p_1	841.8222166	341.6264077	-1.301096085	Down	9.88E-51
miR-133-3p_5	1003.769866	407.5315022	-1.300445052	Down	4.33E-60
miR-2a-3p_6	104.0607015	43.20333962	-1.268210596	Down	2.44E-07
miR-1-3p	63.31915443	27.20210272	-1.218923814	Down	0.000103758
miR-2d_2	50.92693385	22.30172392	-1.191273625	Down	0.000754966
miR-1-3p_4	2679.860138	1197.492566	-1.162141008	Down	1.76E-132
miR-92_2	39.04398262	18.20140697	-1.101050245	Down	0.0046207

Supplementary table. 2 Immune-related miRNA-mRNA target relationships

KEGG term	Gene id	log_2Fold Change	Name of gene	Target region	miRNA	log_2Fold Change
ko04620	PIN_scaffold _5_2562	2.132010317	*IRAK1*	CDS	novel_mir23	-1.484282219
				3UTR	miR-34_5	-3.850817676
					miR-315a	-2.539213904
					miR-71_5	-1.444798087
					novel_mir11	-1.340002067
	PIN_scaffold _9_0911	2.207776338	*CASP8*	CDS	novel_mir1	2.165717009
					miR-71_5	-1.444798087
	PIN_scaffold _10_2490	1.908315817	*TLR1, CD281*	CDS	novel_mir12	-1.911677581
			TLR4, CD284		novel_mir14	-1.673344669
	PIN_scaffold _9_0349	-1.492533177	*FOS*	CDS	novel_mir1	2.165717009
	PIN_scaffold _8_1385	2.432536936	*NFKBIA*	3UTR	miR-7	1.83754921
					let-7-5p_4	1.583938058
					miR-133-3p_5	-1.300445052
	PIN_scaffold _5_0201	2.074249135	*TRAF6*	CDS	miR-34_5	-3.850817676
			TRAF3			
	PIN_scaffold _13_1598	-1.202197825	*ERK, MAPK1_3*	3UTR	miR-315a	-2.539213904
					miR-133-3p_5	-1.300445052
	PIN_scaffold _9_1921	1.180870359	*TRAF3*	3UTR	novel_mir2	-2.02361991
					miR-92_2	-1.101050245
ko00591	PIN_scaffold _7_1936	-2.034777272	*CYP2J*	3UTR	miR-278-3p_6	-1.692096151
					miR-278-3p_1	-1.57520764
	PIN_scaffold _10_0305	-2.163647188	*CYP3A*	CDS	miR-29a_1	-2.020567399
	PIN_scaffold _10_0310	-2.113271407	*CYP3A*	CDS	novel_mir2	-2.02361991
					novel_mir12	-1.911677581
					novel_mir11	-1.340002067
					miR-2d_2	-1.191273625
					miR-92_2	-1.101050245
	PIN_scaffold _8_2110	2.354711321	*PLA2G4, CPLA2*	CDS	novel_mir5	12.52661076

Continuation of the supplementary table 2

KEGG term	Gene id	log_2Fold Change	Name of gene	Target region	miRNA	log_2Fold Change
					miR-2d_2	-1.191273625
	PIN_scaffold_10_1421	-1.871838035	*PLA2G6, IPLA2*	CDS	novel_mir23	-1.484282219
	PIN_scaffold_10_0373	3.089475355	*PLA2G, SPLA2*	CDS	novel_mir2	-2.02361991
					novel_mir12	-1.911677581
					novel_mir14	-1.673344669
					miR-2d_2	-1.191273625
	PIN_scaffold_10_0306	-1.464269909	*CYP3A*	CDS	miR-29a_1	-2.020567399
ko04917	PIN_scaffold_13_1598	-1.202197825	*ERK, MAPK1_3*	3UTR	miR-315a	-2.539213904
					miR-133-3p_5	-1.300445052
	PIN_scaffold_9_1572	-1.706625474	*CYP17A*	CDS	novel_mir22	5.573348613
	PIN_scaffold_9_0349	-1.492533177	*FOS*	CDS	novel_mir1	2.165717009
	PIN_scaffold_10_0809	2.374538324	*SRC*	CDS	miR-34_5	-3.850817676
				3UTR	novel_mir5	12.52661076
					novel_mir1	2.165717009
					miR-278-3p_6	-1.692096151
					miR-278-3p_1	-1.57520764
					miR-2d_2	-1.191273625
ko04624	PIN_scaffold_7_1280	1.549528778	*BIRC2_3*	3UTR	miR-34_5	-3.850817676
	PIN_scaffold_5_2562	2.132010317	*IRAK1*	CDS	novel_mir23	-1.484282219
				3UTR	miR-34_5	-3.850817676
					miR-315a	-2.539213904
					miR-71_5	-1.444798087
					novel_mir11	-1.340002067
	PIN_scaffold_7_2039	2.94333395	*TL*	CDS	miR-34_5	-3.850817676
	PIN_scaffold_10_0914	1.599355517	*TL*	CDS	novel_mir12	-1.911677581
					novel_mir11	-1.340002067
					miR-2a-3p_6	-1.268210596
	PIN_scaffold_7_0207	1.294877634	*BIRC2_3*	CDS	miR-2a-3p_6	-1.268210596

Continuation of the supplementary table 2

KEGG term	Gene id	$\log_2$Fold Change	Name of gene	Target region	miRNA	$\log_2$Fold Change
	PIN_scaffold_8_1385	2.432536936	*NFKBIA*	3UTR	miR-7	1.83754921
					let-7-5p_4	1.583938058
					miR-133-3p_5	-1.300445052
	PIN_scaffold_12_1126	2.337322883	*DUOX, THOX*	CDS	novel_mir22	5.573348613
					miR-133-3p_5	-1.300445052
	PIN_scaffold_7_2434	2.947586276	*DUOX, THOX*	CDS	novel_mir22	5.573348613
					novel_mir12	-1.911677581
					novel_mir14	-1.673344669
					novel_mir11	-1.340002067
					miR-2d_2	-1.191273625
	PIN_scaffold_9_0349	-1.492533177	*FOSLN*	CDS	novel_mir1	2.165717009
	PIN_scaffold_7_1057	2.215863509	*BIRC2_3*	CDS	miR-133-3p_5	-1.300445052
	PIN_scaffold_9_0911	2.207776338	*CASP8*	CDS	novel_mir1	2.165717009
					miR-71_5	-1.444798087
	PIN_scaffold_7_1314	-2.183310091	*MAP3K4, MEKK4*	CDS	miR-71c-5p	7.698124613
					let-7-5p_4	1.583938058
					miR-71_5	-1.444798087
					novel_mir11	-1.340002067
ko00590	PIN_scaffold_7_1936	-2.034777272	*CYP2J*	3UTR	miR-278-3p_6	-1.692096151
			CYP2B		miR-278-3p_1	-1.57520764
	PIN_scaffold_10_0305	-2.163647188	*TBXAS1, CYP5A*	CDS	miR-29a_1	-2.020567399
	PIN_scaffold_10_1421	-1.871838035	*PLA2G6, IPLA2*	CDS	novel_mir23	-1.484282219
	PIN_scaffold_8_2110	2.354711321	*PLA2G4, CPLA2*	CDS	novel_mir5	12.52661076
					miR-2d_2	-1.191273625
	PIN_scaffold_10_0373	3.089475355	*PLA2G, SPLA2*	CDS	novel_mir2	-2.02361991
					novel_mir12	-1.911677581
					novel_mir14	-1.673344669
					miR-2d_2	-1.191273625

Continuation of the supplementary table 2

KEGG term	Gene id	log_2Fold Change	Name of gene	Target region	miRNA	log_2Fold Change
	PIN_scaffold_10_0306	-1.464269909	*TBXAS1, CYP5A*	CDS	miR-29a_1	-2.020567399
	PIN_scaffold_10_0310	-2.113271407	*TBXAS1, CYP5A*	CDS	novel_mir2	-2.02361991
					novel_mir12	-1.911677581
					novel_mir11	-1.340002067
					miR-2d_2	-1.191273625
					miR-92_2	-1.101050245
ko00010	PIN_scaffold_7_1192	-2.51835696	*ALDH*	CDS	novel_mir5	12.52661076
					novel_mir12	-1.911677581
					novel_mir14	-1.673344669
					novel_mir11	-1.340002067
					miR-2d_2	-1.191273625
	PIN_scaffold_12_1710	-2.066883385	*E4.1.1.32, pckA, PCK*	CDS	novel_mir5	12.52661076
					novel_mir14	-1.673344669
					miR-2a-3p_6	-1.268210596
					miR-2d_2	-1.191273625
	PIN_scaffold_6_0946	-1.559614657	*PGK, pgk*	3UTR	novel_mir2	-2.02361991
					miR-8-3p_1	-1.301096085
					miR-92_2	-1.101050245
	PIN_scaffold_11_1147	-1.51185551	*pfkA, PFK*	CDS	miR-29a_1	-2.020567399
					novel_mir12	-1.911677581
					miR-2a-3p_6	-1.268210596
					miR-2d_2	-1.191273625
	PIN_scaffold_12_0085	-1.683393374	*GPI, pgi*	CDS	novel_mir5	12.52661076
					miR-133-3p_5	-1.300445052
	PIN_scaffold_6_0365	-1.647715949	*PGM2*	CDS	miR-34_5	-3.850817676
	PIN_scaffold_10_0852	-1.640091379	*pgm*	3UTR	miR-278-3p_6	-1.692096151
					miR-278-3p_1	-1.57520764
					novel_mir11	-1.340002067
					miR-2a-3p_6	-1.268210596
ko04621	PIN_scaffold_13_1598	-1.202197825	*ERK, MAPK1_3*	3UTR	miR-315a	-2.539213904

Continuation of the supplementary table 2

KEGG term	Gene id	log_2Fold Change	Name of gene	Target region	miRNA	log_2Fold Change
					miR-133-3p_5	-1.300445052
	PIN_scaffold_5_0201	2.074249135	*TRAF6*	CDS	miR-34_5	-3.850817676
			TRAF3			
	PIN_scaffold_8_1385	2.432536936	*NFKBIA*	3UTR	miR-7	1.83754921
					let-7-5p_4	1.583938058
					miR-133-3p_5	-1.300445052
	PIN_scaffold_9_1921	1.180870359	*TRAF3*	3UTR	novel_mir2	-2.02361991
					miR-92_2	-1.101050245
	PIN_scaffold_12_0002	-1.95455919	*NLRC4, CARD12*	CDS	miR-133-3p_5	-1.300445052
			NLRP3, PYPAF1		miR-2d_2	-1.191273625
	PIN_scaffold_9_0911	2.207776338	*CASP8*	CDS	novel_mir1	2.165717009
					miR-71_5	-1.444798087
	PIN_scaffold_7_1057	2.215863509	*BIRC2_3*	CDS	miR-133-3p_5	-1.300445052
	PIN_scaffold_7_0207	1.294877634	*BIRC2_3*	CDS	miR-2a-3p_6	-1.268210596
			XIAP, BIRC4			
	PIN_scaffold_2_0790	-2.934035686	*NLRP12*	CDS	novel_mir11	-1.340002067
					miR-2d_2	-1.191273625
	PIN_scaffold_7_1280	1.549528778	*BIRC2_3*	3UTR	miR-34_5	-3.850817676
	PIN_scaffold_5_2814	2.735577969	*PLCB*	CDS	novel_mir1	2.165717009
	PIN_scaffold_10_2490	1.908315817	*TLR4, CD284*	CDS	novel_mir12	-1.911677581
					novel_mir14	-1.673344669
	PIN_scaffold_13_1147	-1.663978344	*NLRP3, PYPAF1*	3UTR	novel_mir23	-1.484282219
					miR-2d_2	-1.191273625
ko04668	PIN_scaffold_7_0207	1.294877634	*BIRC2_3*	CDS	miR-2a-3p_6	-1.268210596
			XIAP, BIRC4			

Continuation of the supplementary table 2

KEGG term	Gene id	log2Fold Change	Name of gene	Target region	miRNA	log2Fold Change
	PIN_scaffold_9_0911	2.207776338	*CASP10*	CDS	novel_mir1	2.165717009
			CASP8		miR-71_5	-1.444798087
	PIN_scaffold_8_1385	2.432536936	*NFKBIA*	3UTR	miR-7	1.83754921
					let-7-5p_4	1.583938058
					miR-133-3p_5	-1.300445052
	PIN_scaffold_13_1223	-2.126744978	*MMP14*	CDS	novel_mir11	-1.340002067
					miR-2d_2	-1.191273625
	PIN_scaffold_5_0201	2.074249135	*TRAF3*	CDS	miR-34_5	-3.850817676
	PIN_scaffold_7_1028	1.390524203	*JAG1, CD339*	CDS	miR-34_5	-3.850817676
	PIN_scaffold_9_0349	-1.492533177	*FOS*	CDS	novel_mir1	2.165717009
	PIN_scaffold_7_1280	1.549528778	*BIRC2_3*	3UTR	miR-34_5	-3.850817676
	PIN_scaffold_13_1598	-1.202197825	*ERK, MAPK1_3*	3UTR	miR-315a	-2.539213904
					miR-133-3p_5	-1.300445052
	PIN_scaffold_9_1921	1.180870359	*TRAF3*	3UTR	novel_mir2	-2.02361991
					miR-92_2	-1.101050245
	PIN_scaffold_7_1057	2.215863509	*BIRC2_3*	CDS	miR-133-3p_5	-1.300445052
ko04064	PIN_scaffold_7_1280	1.549528778	*BIRC2_3*	3UTR	miR-34_5	-3.850817676
	PIN_scaffold_5_0201	2.074249135	*TRAF6*	CDS	miR-34_5	-3.850817676
			TRAF3			
	PIN_scaffold_7_0207	1.294877634	*BIRC2_3*	CDS	miR-2a-3p_6	-1.268210596
			XIAP, BIRC4			
	PIN_scaffold_8_1385	2.432536936	*NFKBIA*	3UTR	miR-7	1.83754921
					let-7-5p_4	1.583938058
					miR-133-3p_5	-1.300445052
	PIN_scaffold_7_1057	2.215863509	*BIRC2_3*	CDS	miR-133-3p_5	-1.300445052

Continuation of the supplementary table 2

KEGG term	Gene id	log_2Fold Change	Name of gene	Target region	miRNA	log_2Fold Change
	PIN_scaffold_9_1921	1.180870359	*TRAF3*	3UTR	novel_mir2	-2.02361991
					miR-92_2	-1.101050245
	PIN_scaffold_12_1302	3.660867558	*TRIM25, EFP*	CDS	miR-92_2	-1.101050245
	PIN_scaffold_5_2562	2.132010317	*IRAK1*	CDS	novel_mir23	-1.484282219
				3UTR	miR-34_5	-3.850817676
					miR-315a	-2.539213904
					miR-71_5	-1.444798087
					novel_mir11	-1.340002067
	PIN_scaffold_6_0659	3.369499044	*TRIM25, EFP*	CDS	novel_mir5	12.52661076
					novel_mir23	-1.484282219
	PIN_scaffold_10_2490	1.908315817	*TLR4, CD284*	CDS	novel_mir12	-1.911677581
					novel_mir14	-1.673344669
	PIN_scaffold_14_0204	-2.234370194	*TRIM25, EFP*	CDS	novel_mir22	5.573348613
					novel_mir12	-1.911677581
					miR-278-3p_6	-1.692096151
					miR-278-3p_1	-1.57520764
					miR-2a-3p_6	-1.268210596
	PIN_scaffold_12_1551	-1.613195281	*DDX58, RIG-I*	CDS	novel_mir5	12.52661076
	PIN_scaffold_3_0002	2.209532221	*TRIM25, EFP*	CDS	novel_mir23	-1.484282219
ko04146	PIN_scaffold_8_0609	2.133980055	*SOD1*	CDS	miR-92_2	-1.101050245
					novel_mir2	-2.02361991
					novel_mir23	-1.484282219

Continuation of the supplementary table 2

Supplementary Table.3 Expression of target genes interacting with miRNAs

	average_fpkm_PBS	average_fpkm_LPS	diffexp_log2fc_PBS-vs-LPS	diffexp_deseq2_qvalue_PBS-vs-LPS	diffexp_pvalue_PBS-vs-LPS
PIN_scaffold_8_0660	1.65	7.23	2.407358614	0.006355042	0.000160516
PIN_scaffold_5_2562	34.06	113.436	2.132010317	0.006133492	0.000153319
PIN_scaffold_2_0306	7.616	33.56	2.390509907	0.000124281	1.29E-06
PIN_scaffold_7_1799	0.343	7.996	4.351202436	6.12E-08	2.11E-10
PIN_scaffold_7_2505	48.186	162.28	2.175964438	1.96E-05	1.44E-07
PIN_scaffold_10_1431	13.776	21.506	1.051395735	0.045958067	0.002326355
PIN_scaffold_3_0427	24.473	70.266	1.867386633	0.000428268	5.97E-06
PIN_scaffold_7_0277	0.55	5.14	3.417772566	4.63E-07	2.15E-09
PIN_scaffold_5_3197	4.263	18.7	2.678254296	2.80E-10	6.90E-13
PIN_scaffold_10_0782	7.653	36.066	2.515896373	0.000261425	3.16E-06
PIN_scaffold_6_1615	8.55	20.136	1.594659625	0.0088429	0.000249368
PIN_scaffold_8_0797	62.506	302.376	2.580112279	7.08E-06	4.37E-08
PIN_scaffold_12_1338	24.776	71.93	1.927412634	0.005540091	0.000134591
PIN_scaffold_7_0439	0	5.476	4.555548183	0.000609838	9.29E-06
PIN_scaffold_11_0575	248.326	823.996	1.940056612	0.018865207	0.000675678
PIN_scaffold_11_0522	6.773	9.22	1.215590915	0.034739375	0.00154545
PIN_scaffold_4_0948	20.51	40.986	1.279557544	0.04125972	0.002001507
PIN_scaffold_9_1730	0.223	5.323	3.668342232	0.004124127	9.24E-05

PIN_scaffold_8_0609	4.456	15.196	2.133980055	0.000285732	3.57E-06
PIN_scaffold_7_1118	15.3	84.726	2.813263939	4.08E-06	2.36E-08
PIN_scaffold_5_3227	0.5	8.02	3.414101608	0.004424565	0.000102306
PIN_scaffold_6_0430	0.61	11.716	3.413184311	0.007243424	0.000192381
PIN_scaffold_1_1158	11.603	37.593	2.041772776	0.000574337	8.66E-06
PIN_scaffold_6_1172	0.04	2.886	4.694898448	7.61E-05	7.36E-07
PIN_scaffold_8_0720	2.416	7.196	1.894854122	0.001920665	3.71E-05
PIN_scaffold_11_0964	10.713	37.803	2.115648019	0.002498112	5.12E-05
PIN_scaffold_13_1302	2.313	34.9	4.026255678	2.32E-10	5.53E-13
PIN_scaffold_8_2110	0.28	1.286	2.354711321	0.029419242	0.001240979
PIN_scaffold_5_0401	0.026	3.15	5.12475037	1.24E-05	8.28E-08
PIN_scaffold_10_0101	5.72	39.256	3.02187221	1.08E-07	3.99E-10
PIN_scaffold_7_0053	1.733	184.373	3.51682889	0.023124803	0.000888755
PIN_scaffold_13_0178	0.136	2.483	3.80786465	0.000270151	3.32E-06
PIN_scaffold_9_0785	4.29	9.033	1.378808772	0.047260444	0.002418122
PIN_scaffold_3_0004	0.03	0.18	2.563250005	0.044535221	0.00223377
PIN_scaffold_5_0240	0.65	6.286	3.537567654	2.70E-10	6.53E-13
PIN_scaffold_8_1871	7.753	33.93	2.366879535	0.004301677	9.78E-05
PIN_scaffold_11_0400	1.74	4.653	1.960549494	0.014546885	0.000487491
PIN_scaffold_10_1691	7.476	26.243	2.097109454	0.000366855	4.97E-06
PIN_scaffold_8_0034	7.493	26.203	2.003059892	0.044535221	0.002235197
PIN_scaffold_10_0633	1.176	21.4	3.592474196	0.000787269	1.29E-05

PIN_scaffold_13_1022	2.37	9.453	2.22702804	0.005867933	0.000144618
PIN_scaffold_13_0016	922.016	1628.176	1.194773441	0.04067758	0.001948648
PIN_scaffold_14_0465	0.263	30.063	3.377287807	0.034447235	0.001525726
PIN_scaffold_9_1584	82.416	1187.053	4.150027306	3.78E-19	2.21E-22
PIN_scaffold_11_0501	0.393	2.39	2.610804194	0.027966525	0.001153338
PIN_scaffold_1_0715	1.576	8.87	2.52132801	0.016227873	0.000566008
PIN_scaffold_5_3201	4.726	13.743	1.871680284	0.005557992	0.000135243
PIN_scaffold_7_0938	4.773	10.203	1.411437482	0.031542777	0.001369979
PIN_scaffold_4_1028	1.43	3.936	1.799061798	0.006715939	0.0001726
PIN_scaffold_14_0064	9.783	43.77	2.564629103	5.34E-06	3.23E-08
PIN_scaffold_6_0081	0.62	9.31	3.447936313	0.000676218	1.07E-05
PIN_scaffold_8_0124	76.473	544.33	3.103242099	4.02E-07	1.82E-09
PIN_scaffold_1_0983	0.68	15.4	4.80168833	5.30E-25	4.14E-29
PIN_scaffold_8_2018	82.153	141.586	1.186712856	0.031386126	0.001357247
PIN_scaffold_12_1302	6.196	84.766	3.660867558	1.86E-05	1.36E-07
PIN_scaffold_14_0119	14.076	45.29	1.933955597	0.020922542	0.000778761
PIN_scaffold_10_1468	18.53	55.76	1.985863139	0.000126494	1.33E-06
PIN_scaffold_11_0196	3.89	8.98	1.434856219	0.012219421	0.000378996
PIN_scaffold_11_0683	0.573	1.95	2.061426057	0.021552408	0.000811488
PIN_scaffold_10_1432	35.5	87.913	1.786746511	0.035565317	0.001600252
PIN_scaffold_8_1262	0.2	1.726	2.782302261	0.023777775	0.000916637
PIN_scaffold_12_1083	175.12	282.3	1.148422468	0.012721131	0.000402954

PIN_scaffold_5_2023	22.43	51.52	1.654965033	0.0211097	0.000787398
PIN_scaffold_10_2490	1.033	3.526	1.908315817	0.033144396	0.001451192
PIN_scaffold_6_0327	56.87	99.623	1.265021531	0.007622031	0.000206386
PIN_scaffold_5_1401	0.183	5.343	4.069006408	0.000360356	4.83E-06
PIN_scaffold_10_1567	1.006	5.27	2.384293355	0.028098495	0.001166609
PIN_scaffold_1_0952	0.2	1.253	2.887280558	0.036418631	0.00165856
PIN_scaffold_10_2973	15.286	38.48	1.680309961	0.000657437	1.04E-05
PIN_scaffold_3_0706	13.06	32.783	1.69845101	0.000408367	5.68E-06
PIN_scaffold_1_0832	8.873	49.566	2.853956851	1.08E-07	4.01E-10
PIN_scaffold_11_1782	1.283	2.51	1.370473032	0.046406527	0.00235993
PIN_scaffold_11_0572	0.97	15.523	3.219656455	0.018525105	0.000661494
PIN_scaffold_10_2500	3.53	8.963	1.767480402	0.001213054	2.17E-05
PIN_scaffold_5_1089	22.246	54.293	1.689501999	0.006085381	0.000151641
PIN_scaffold_7_2221	3.45	17.423	2.107747443	0.001445906	2.65E-05
PIN_scaffold_9_1427	7.293	30.31	2.164950534	0.037152887	0.001703609
PIN_scaffold_3_0192	16.54	28.516	1.265651415	0.006586002	0.000168232
PIN_scaffold_14_0112	1.546	10.936	2.944251407	1.75E-05	1.26E-07
PIN_scaffold_1_0417	3.636	57.77	4.173894651	1.23E-11	2.26E-14
PIN_scaffold_12_1227	4.846	8.453	1.19799873	0.030342161	0.001296473
PIN_scaffold_8_1941	0.66	3.606	2.690879128	0.002450151	4.99E-05
PIN_scaffold_5_2814	0.396	3.446	2.735577969	0.036521324	0.00166609
PIN_scaffold_4_0394	14.953	33.663	1.735763372	0.0148886	0.000503815

PIN_scaffold_12_0194	60.426	120.806	1.509428071	0.005209034	0.000125124
PIN_scaffold_5_2137	0.736	39.356	5.754067899	6.42E-19	4.27E-22
PIN_scaffold_9_1796	3.673	8.913	1.661223863	0.039667084	0.00187467
PIN_scaffold_10_1870	1.696	8.046	2.544289558	0.008680712	0.000244455
PIN_scaffold_10_2585	60.466	167.14	1.941248586	0.007003172	0.000184085
PIN_scaffold_12_1344	0.41	1.65	2.298779664	0.00753371	0.000203033
PIN_scaffold_10_2707	601.6	1129	1.375579998	0.006674799	0.000171022
PIN_scaffold_13_1094	32	104.6	2.249624189	0.037272563	0.001710552
PIN_scaffold_10_0052	0.98	4.713	2.453325262	0.034961976	0.001564911
PIN_scaffold_9_0032	0.486	3.996	3.092896443	6.45E-05	6.02E-07
PIN_scaffold_2_0423	0.733	4.316	2.459293861	0.015937489	0.000551522
PIN_scaffold_8_1430	8.603	34.146	2.466143647	0.008166261	0.000225176
PIN_scaffold_3_0002	0.683	4.106	2.209532221	0.030609401	0.001312703
PIN_scaffold_14_0584	6.203	24.52	2.554711464	1.11E-07	4.20E-10
PIN_scaffold_10_1047	51.116	771.76	4.040174276	4.09E-08	1.33E-10
PIN_scaffold_5_2300	24.883	94.276	2.296057224	0.003867428	8.56E-05
PIN_scaffold_12_0653	13.86	90.843	2.584839295	0.000599054	9.08E-06
PIN_scaffold_7_1028	73.99	144.42	1.390524203	0.008516006	0.000238154
PIN_scaffold_8_1986	0.923	3.503	2.233047615	0.002018914	3.94E-05
PIN_scaffold_11_1950	0	0.356	4.193890865	0.00245728	5.01E-05
PIN_scaffold_5_3310	2.41	10.876	2.332847699	0.00170293	3.22E-05
PIN_scaffold_3_0521	0.353	7.883	3.628662737	0.002071564	4.08E-05

PIN_scaffold_10_3227	4.753	6.923	1.150410095	0.02463128	0.000959161
PIN_scaffold_8_0798	0.24	2.016	3.554899235	2.45E-05	1.91E-07
PIN_scaffold_10_3220	0.89	2.643	1.888636235	0.046057499	0.002335461
PIN_scaffold_10_0809	210.576	848.646	2.374538324	1.67E-05	1.19E-07
PIN_scaffold_7_1255	2.236	7.05	2.015884156	0.003391209	7.32E-05
PIN_scaffold_12_0392	17.233	33.91	1.551321624	0.001148532	2.03E-05
PIN_scaffold_10_2554	10.306	21.36	1.509981868	0.011789818	0.000355956
PIN_scaffold_12_0152	14.156	25.443	1.314788516	0.024165402	0.000932524
PIN_scaffold_8_0121	2.18	10.823	2.471975309	0.000982615	1.67E-05
PIN_scaffold_8_0202	0.836	3.02	2.184120417	0.001473213	2.72E-05
PIN_scaffold_5_3304	63.686	225.71	2.192552154	7.85E-07	3.83E-09
PIN_scaffold_10_2167	18.106	115.136	2.776050299	0.00071994	1.16E-05
PIN_scaffold_3_0779	7.476	17.086	1.580440619	0.013462613	0.000436958
PIN_scaffold_12_0473	0.073	11.416	4.204601758	0.001901637	3.66E-05
PIN_scaffold_11_0977	20.266	44.983	1.50255256	0.005849788	0.000143746
PIN_scaffold_8_1895	27.716	67.343	1.716888948	0.002021997	3.96E-05
PIN_scaffold_3_0698	1.59	3.906	1.577037741	0.041886256	0.002049896
PIN_scaffold_12_0525	9	26.31	1.959861963	0.000315253	4.06E-06
PIN_scaffold_5_0323	2.023	10.516	2.392895009	0.020922542	0.000778782
PIN_scaffold_5_0501	79.27	175.586	1.609445386	0.046057499	0.002336032
PIN_scaffold_5_0201	119.226	384.746	2.074249135	3.90E-05	3.35E-07
PIN_scaffold_3_0388	1.7	17.87	3.514739427	3.16E-05	2.55E-07

PIN_scaffold_2_0572	0.453	10.4	3.435327082	0.010438923	0.0003062
PIN_scaffold_11_0071	2.896	12.193	2.480856056	4.36E-05	3.87E-07
PIN_scaffold_8_0859	0.18	0.883	2.304876814	0.0324384	0.001412679
PIN_scaffold_10_2857	7.096	12.303	1.138771002	0.040365309	0.001923434
PIN_scaffold_5_0256	1.37	4.58	2.093900285	0.00444745	0.000103183
PIN_scaffold_7_1057	10.19	37.666	2.215863509	0.014737865	0.000496747
PIN_scaffold_9_1012	74.043	160.073	1.728735916	0.001103597	1.94E-05
PIN_scaffold_1_0947	1.333	5.786	2.199979577	0.028459792	0.001187168
PIN_scaffold_13_1027	81.27	145.083	1.258843123	0.003843487	8.50E-05
PIN_scaffold_10_0647	0.013	2.096	4.329344223	0.001271711	2.29E-05
PIN_scaffold_5_1356	28.546	117.283	2.267994532	0.000156523	1.74E-06
PIN_scaffold_9_0605	3.066	12.846	2.388724634	0.00086154	1.42E-05
PIN_scaffold_7_2039	41.243	285.683	2.94333395	0.005125471	0.000122516
PIN_scaffold_10_2257	0.183	1.083	2.467543207	0.045330693	0.002280433
PIN_scaffold_5_2902	2.073	16.006	3.037666545	2.65E-05	2.08E-07
PIN_scaffold_5_1827	193.803	419.04	1.54902281	0.01604273	0.000556417
PIN_scaffold_12_0283	57.106	102.59	1.256966602	0.019180282	0.000688461
PIN_scaffold_5_0605	117.496	385.946	2.106170374	1.73E-05	1.23E-07
PIN_scaffold_5_3143	1.916	11.28	2.477904666	0.019999413	0.000727237
PIN_scaffold_7_0207	28.27	51.06	1.294877634	0.041743464	0.002039647
PIN_scaffold_3_0081	1.806	3.766	1.553308519	0.040680543	0.001951468
PIN_scaffold_7_1945	18.783	32.41	1.26645833	0.015427442	0.000529654

PIN_scaffold_8_0719	15.83	45.34	1.841545068	0.007960833	0.000217654
PIN_scaffold_10_0157	0.11	4.67	6.369294195	2.94E-15	3.33E-18
PIN_scaffold_7_2553	0.013	0.896	3.451487069	0.021611627	0.00081625
PIN_scaffold_11_1343	57.086	153.05	1.836563641	2.38E-05	1.84E-07
PIN_scaffold_8_1607	9.713	20.076	1.454420931	0.037406502	0.001722543
PIN_scaffold_8_1385	4.756	19.42	2.432536936	0.000386703	5.35E-06
PIN_scaffold_7_2434	3.443	23.42	2.947586276	2.25E-05	1.71E-07
PIN_scaffold_10_1853	12.336	131.9	3.694832545	1.13E-10	2.39E-13
PIN_scaffold_7_2302	0.416	5.536	2.965814515	0.041032052	0.001976039
PIN_scaffold_6_0659	0.156	1.983	3.369499044	0.001773557	3.37E-05
PIN_scaffold_6_1338	0.276	2.833	3.259898131	0.000497527	7.23E-06
PIN_scaffold_5_3450	2.863	9.196	2.039203883	0.001552642	2.90E-05
PIN_scaffold_5_0049	12.996	83.97	3.19123284	6.08E-09	1.76E-11
PIN_scaffold_10_0051	5.043	12.363	1.693835004	0.022812862	0.000875876
PIN_scaffold_13_0601	0.33	2.266	2.832308947	0.041141524	0.001994166
PIN_scaffold_7_2137	32.093	58.686	1.167493746	0.030558194	0.001309313
PIN_scaffold_10_0672	9.943	90.606	3.705161885	1.07E-14	1.34E-17
PIN_scaffold_14_0605	2.08	12.696	2.830082123	0.000787269	1.29E-05
PIN_scaffold_13_0964	1.11	10.663	3.078762215	0.002613066	5.41E-05
PIN_scaffold_8_1042	51.3	114.046	1.566197588	0.00438894	0.000100794
PIN_scaffold_5_2573	115.213	425.406	2.284682704	9.54E-05	9.50E-07
PIN_scaffold_10_0573	0.183	1.586	2.889704574	0.01448976	0.000484445

PIN_scaffold_10_2669	63.606	146.983	1.747035581	0.001237167	2.22E-05
PIN_scaffold_2_0814	15.96	54.786	2.229329445	0.000248526	2.97E-06
PIN_scaffold_9_0911	1.223	4.73	2.207776338	0.012505729	0.0003932
PIN_scaffold_5_3093	23.606	60.28	1.666374736	0.012786758	0.000406452
PIN_scaffold_9_1805	1.386	3.9	1.753322078	0.040674427	0.0019439
PIN_scaffold_10_1881	5.636	15.093	1.848979277	0.04695977	0.002397235
PIN_scaffold_8_0628	75.646	141.196	1.342398533	0.011905336	0.000362836
PIN_scaffold_13_0349	31.013	59.85	1.387146078	0.029549071	0.001252226
PIN_scaffold_5_2927	29.406	81.383	1.868974394	0.005434275	0.000131384
PIN_scaffold_13_0792	13.433	37.703	1.567516129	0.010068937	0.000292987
PIN_scaffold_3_0970	1.113	3.673	2.052189835	0.013049188	0.000419462
PIN_scaffold_2_0801	0.24	1.236	2.534890459	0.006475136	0.000164529
PIN_scaffold_10_1048	98.33	1289.89	3.862695685	2.06E-10	4.84E-13
PIN_scaffold_10_0371	190.46	847.736	2.41260684	0.000123653	1.27E-06
PIN_scaffold_5_0874	2.99	39.436	3.768724912	4.07E-06	2.34E-08
PIN_scaffold_11_1783	2.26	9.523	2.230158459	0.000968731	1.64E-05
PIN_scaffold_7_0349	13.43	26.9	1.516262443	0.006734969	0.000173681
PIN_scaffold_5_3010	1.763	13.486	2.997383712	0.000257231	3.09E-06
PIN_scaffold_13_1208	2.896	6.923	1.587762392	0.015119838	0.000517321
PIN_scaffold_10_0194	0.343	33.79	3.475152701	0.025986189	0.001038311
PIN_scaffold_9_0455	0.003	0.166	3.745340425	0.008334761	0.000230807
PIN_scaffold_13_0133	15.043	81.33	2.768565358	1.01E-06	5.12E-09

PIN_scaffold_7_0330	0	4.25	4.746672753	0.000287832	3.62E-06
PIN_scaffold_5_0538	144.396	363.646	1.758326775	0.000949941	1.59E-05
PIN_scaffold_4_0470	0.126	1.043	3.017157351	0.001003663	1.72E-05
PIN_scaffold_5_2830	318.433	783.44	1.695148331	0.021695922	0.000821115
PIN_scaffold_10_2672	42.913	88.646	1.45496496	0.003191248	6.83E-05
PIN_scaffold_3_0038	10.58	21.473	1.437846215	0.013158293	0.00042432
PIN_scaffold_7_2254	119.553	245.633	1.516008623	0.006870254	0.000178981
PIN_scaffold_9_1838	63.763	141.68	1.485238323	0.039482517	0.001858237
PIN_scaffold_5_1651	0.293	13.11	3.410720428	0.005641161	0.000137928
PIN_scaffold_8_1894	11.103	28.083	1.646289546	0.008457089	0.000235529
PIN_scaffold_4_0718	0.196	2.1	3.089894812	0.008257155	0.000228335
PIN_scaffold_12_0082	0.353	4.486	3.311160675	0.006355042	0.000160595
PIN_scaffold_6_0322	165.306	1170.776	3.108406453	3.16E-05	2.55E-07
PIN_scaffold_5_0395	44.36	451.99	3.645396292	2.97E-12	4.98E-15
PIN_scaffold_3_0334	33.743	90.253	1.700801708	0.028049319	0.001163472
PIN_scaffold_11_0031	4.423	11.07	1.718128352	0.000553985	8.27E-06
PIN_scaffold_10_0504	17.89	48.843	1.908870468	0.002498112	5.11E-05
PIN_scaffold_9_0320	83.123	132.333	1.131489985	0.013760953	0.00044938
PIN_scaffold_5_0507	70.37	139.176	1.402767078	0.012610932	0.000398689
PIN_scaffold_9_1921	10.743	17.883	1.180870359	0.029195128	0.001228666
PIN_scaffold_3_1167	0.096	20.956	3.491593131	0.026621853	0.001068909
PIN_scaffold_10_1260	0.08	2.69	4.081502718	0.000574208	8.63E-06

PIN_scaffold_9_0582	0.143	16.46	3.496845626	0.025029065	0.000987234
PIN_scaffold_4_0069	0.033	1.25	3.595575848	0.010683253	0.000317875
PIN_scaffold_6_0612	1.716	9.72	2.831456606	1.42E-05	9.71E-08
PIN_scaffold_10_1252	0.513	5.62	2.813871551	0.041679305	0.002033256
PIN_scaffold_11_1878	15.976	71.836	2.451524705	0.001459777	2.69E-05
PIN_scaffold_14_0294	187.383	381.296	1.423030276	0.001581419	2.96E-05
PIN_scaffold_2_0236	1.486	12.276	2.667651712	0.039969403	0.001896763
PIN_scaffold_9_0460	5.323	45.946	3.11276896	0.000115062	1.17E-06
PIN_scaffold_5_2571	17.656	122	3.071393579	5.90E-11	1.20E-13
PIN_scaffold_7_2507	57.503	137.97	1.717547188	0.025029065	0.000989314
PIN_scaffold_14_1097	6.79	15.146	1.514975218	0.008971834	0.000255807
PIN_scaffold_8_0839	11.78	19.966	1.119298595	0.0148886	0.000504757
PIN_scaffold_12_1764	3.15	539.723	6.406823479	1.68E-13	2.30E-16
PIN_scaffold_8_1916	9.896	22.883	1.7503584	0.000160426	1.79E-06
PIN_scaffold_10_0172	46.416	833.963	4.047466893	1.87E-08	5.71E-11
PIN_scaffold_8_0376	2.756	7.266	1.675812917	0.020922542	0.000777868
PIN_scaffold_11_0900	0.936	4.066	2.075500637	0.028240513	0.001175815
PIN_scaffold_10_0165	0.05	6.706	3.226544444	0.049510389	0.002600925
PIN_scaffold_5_0080	2.13	11.673	2.634418482	0.000307986	3.95E-06
PIN_scaffold_11_1680	2.74	14.373	2.414437586	0.006914961	0.000181226
PIN_scaffold_6_1277	2.743	13.06	2.749460719	0.003311972	7.13E-05
PIN_scaffold_12_0467	1.856	15.496	2.652519039	0.014397313	0.000480229

PIN_scaffold_5_1515	1.9	7.016	2.053508705	0.049083753	0.002559341
PIN_scaffold_10_1702	58.416	410.703	3.052151735	6.47E-09	1.90E-11
PIN_scaffold_3_1095	38.25	105.99	1.86499219	0.000250314	3.00E-06
PIN_scaffold_8_1347	16.123	32.336	2.243358616	1.29E-07	5.10E-10
PIN_scaffold_10_1375	170.84	661.52	2.342977513	4.34E-05	3.83E-07
PIN_scaffold_4_1031	0.846	9.046	3.170961316	0.006028868	0.000149762
PIN_scaffold_5_1422	9.21	17.533	1.420592914	0.0148886	0.00050475
PIN_scaffold_10_1434	3.316	27.726	3.070527358	0.000246537	2.94E-06
PIN_scaffold_5_3160	12.62	21.613	1.215268996	0.006391445	0.000161764
PIN_scaffold_14_0347	0.32	1.343	2.28908263	0.007591055	0.000204875
PIN_scaffold_8_1818	0.393	2.433	2.567419223	0.039185058	0.001839645
PIN_scaffold_12_1787	19.923	39.933	1.492570396	0.031438106	0.001364205
PIN_scaffold_8_0192	2.13	18.016	2.955196408	0.000644759	1.01E-05
PIN_scaffold_5_2497	0	2.153	3.35746145	0.036682722	0.001679184
PIN_scaffold_10_1675	30.2	242.05	3.35593407	1.47E-17	1.15E-20
PIN_scaffold_11_1091	258.303	1167.426	2.523146348	5.55E-09	1.58E-11
PIN_scaffold_1_0533	2.593	8.18	1.774677944	0.03883312	0.001809472
PIN_scaffold_9_1312	0.703	2.68	2.162310446	0.020112314	0.000733699
PIN_scaffold_5_2501	2.8	6.26	1.568074294	0.036357856	0.001652953
PIN_scaffold_14_0281	12.253	25.566	1.473117759	0.042644164	0.002113637
PIN_scaffold_10_0133	16.256	34.48	1.490378754	0.004351868	9.93E-05
PIN_scaffold_4_0245	0.49	8.326	3.542014637	0.001896979	3.65E-05

PIN_scaffold_3_0819	21.466	41.61	1.372786845	0.002839937	5.95E-05
PIN_scaffold_10_2986	45.516	86.263	1.47041671	0.017568515	0.000621002
PIN_scaffold_5_1458	7.573	25.346	2.115836105	0.000309868	3.98E-06
PIN_scaffold_10_2928	1.463	7.12	2.541701602	6.86E-05	6.49E-07
PIN_scaffold_9_1582	61.95	180.903	1.809861183	0.001191293	2.12E-05
PIN_scaffold_1_0858	0.586	3.056	2.530214675	0.022119374	0.000838883
PIN_scaffold_3_0891	22.516	37.1	1.186274148	0.033107249	0.001448272
PIN_scaffold_6_1367	5.13	10.226	1.371354646	0.029700253	0.001260953
PIN_scaffold_2_0299	165.743	429.19	1.819336282	0.027176439	0.001101529
PIN_scaffold_10_0233	0.55	2.133	2.227681565	0.003191248	6.83E-05
PIN_scaffold_4_0774	0.29	3.963	3.608589756	0.000213643	2.47E-06
PIN_scaffold_13_0720	0.773	16.586	4.677549822	7.90E-14	1.05E-16
PIN_scaffold_2_1255	0.226	0.923	2.219757616	0.043563963	0.002176241
PIN_scaffold_9_1025	79.253	145.756	1.295404144	0.015621708	0.000537543
PIN_scaffold_10_2638	56.45	147.036	1.749296146	4.43E-05	3.95E-07
PIN_scaffold_13_0534	0.603	11.7	4.070706665	4.44E-06	2.58E-08
PIN_scaffold_14_0750	36.39	69.84	1.432121496	0.002964153	6.25E-05
PIN_scaffold_14_0002	6.033	45.226	3.112692595	2.50E-06	1.41E-08
PIN_scaffold_9_0544	13.97	24.69	1.151494004	0.026816143	0.001081946
PIN_scaffold_5_3312	7.146	15.603	1.551947439	0.004443218	0.000102911
PIN_scaffold_13_1292	8.906	18.593	1.432718416	0.043619319	0.00218071
PIN_scaffold_10_0914	33.526	75.23	1.599355517	0.002947734	6.20E-05

PIN_scaffold_10_1430	6.866	36.153	2.35312965	0.02258686	0.00086367
PIN_scaffold_7_0092	1.526	5.963	2.17301934	0.019718568	0.000714714
PIN_scaffold_9_0339	1.756	9.816	2.437429499	0.021590545	0.000813767
PIN_scaffold_8_0739	74.956	114.983	1.073444514	0.009905239	0.00028745
PIN_scaffold_7_0217	5.7	18.483	1.823840223	0.040596808	0.001937636
PIN_scaffold_3_0650	11.276	112.156	3.416768887	2.68E-07	1.16E-09
PIN_scaffold_10_0373	0.713	6.22	3.089475355	0.000644759	1.01E-05
PIN_scaffold_6_0678	1.086	5.13	2.425991212	0.001843469	3.53E-05
PIN_scaffold_7_1531	15.026	64.18	2.384597608	3.26E-05	2.69E-07
PIN_scaffold_9_1256	646.35	1164.4	1.326999743	0.014737865	0.000497033
PIN_scaffold_10_0350	441.276	732.57	1.11140462	0.045746973	0.002311067
PIN_scaffold_11_0574	23.656	136.396	2.97454043	1.55E-06	8.37E-09
PIN_scaffold_9_0194	17.713	42.856	1.651502824	0.026816143	0.001077978
PIN_scaffold_9_2033	16.846	78.093	2.337417982	0.004409212	0.000101434
PIN_scaffold_10_3198	647.803	1281.176	1.323364694	0.036265139	0.001644488
PIN_scaffold_3_0348	15.903	41.48	1.74046702	0.001959493	3.81E-05
PIN_scaffold_5_2233	241.116	1727.136	3.134397581	2.84E-07	1.24E-09
PIN_scaffold_5_1325	0.52	4.506	2.986892703	0.013934394	0.000458801
PIN_scaffold_10_2984	34.326	62.256	1.237188523	0.04770458	0.002455753
PIN_scaffold_3_0955	25.693	47.693	1.33746391	0.000620804	9.60E-06
PIN_scaffold_14_0387	70.71	106.593	1.410526743	0.022148813	0.000840871
PIN_scaffold_10_2055	8.146	33.773	2.369720116	1.74E-06	9.44E-09

PIN_scaffold_8_1731	2.883	14.59	2.624697718	2.05E-08	6.32E-11
PIN_scaffold_4_0464	1.936	34.266	4.181070422	1.33E-10	2.91E-13
PIN_scaffold_13_1066	1.55	4.6	1.929567286	0.023556987	0.000907206
PIN_scaffold_9_0627	4.03	39.403	3.254615079	3.74E-08	1.20E-10
PIN_scaffold_9_1366	0.016	0.833	4.004214543	0.001584947	2.98E-05
PIN_scaffold_11_0370	177.493	437.76	1.713515551	0.01777321	0.00063032
PIN_scaffold_12_0018	10.05	25.143	1.882957361	0.04244988	0.002093913
PIN_scaffold_5_1284	14.906	143.776	3.412421998	4.63E-07	2.17E-09
PIN_scaffold_9_0461	2.78	21.746	2.76393777	0.00073284	1.19E-05
PIN_scaffold_8_1001	41.73	77.246	1.199792915	0.049486366	0.00259773
PIN_scaffold_9_0458	0.843	11.836	3.942519241	1.22E-06	6.28E-09
PIN_scaffold_5_3019	3.596	8.853	1.74693142	0.001058452	1.83E-05
PIN_scaffold_11_0997	30.786	174.076	2.789171125	4.22E-09	1.19E-11
PIN_scaffold_3_0769	0.26	1.963	2.904195464	0.01458358	0.00048929
PIN_scaffold_5_2346	2.116	5.58	1.713566473	0.039973273	0.001898508
PIN_scaffold_7_1494	3.083	9.243	1.883722304	0.01125356	0.000336688
PIN_scaffold_6_0657	41.676	143.66	2.135795993	0.0003506	4.67E-06
PIN_scaffold_7_2359	0.07	3.823	4.540063727	9.86E-05	9.86E-07
PIN_scaffold_11_0979	3.973	12.076	2.014246922	8.44E-06	5.47E-08
PIN_scaffold_10_1687	0.41	4.63	3.044869627	0.012786758	0.000406531
PIN_scaffold_7_1777	4.623	10.136	1.899428446	0.024403334	0.000945007
PIN_scaffold_12_0527	5.936	13.723	1.574445445	0.008478058	0.000236762

PIN_scaffold_4_0537	1.356	4.16	1.943113649	0.001376033	2.49E-05
PIN_scaffold_7_0812	1.42	14.333	2.882475873	0.048631511	0.002524363
PIN_scaffold_11_0795	33.43	227.886	3.101977613	8.75E-08	3.04E-10
PIN_scaffold_13_0996	1.626	4.493	1.957788063	0.008142677	0.000223261
PIN_scaffold_5_2516	15.096	33.023	1.521713083	0.024857093	0.00097475
PIN_scaffold_9_1216	5.896	101.743	4.321948352	5.78E-16	5.42E-19
PIN_scaffold_14_0262	35.98	55.183	1.033667271	0.037406502	0.001721828
PIN_scaffold_8_1370	0.26	1.696	2.528911666	0.045417175	0.002286558
PIN_scaffold_5_0068	0.136	4.746	3.602274546	0.010560322	0.000310586
PIN_scaffold_8_0866	1.806	5.683	1.916788124	0.009424192	0.000270178
PIN_scaffold_5_1369	6.796	89.986	3.533588971	4.45E-05	3.99E-07
PIN_scaffold_7_0332	98.633	235.123	1.680360084	0.011905336	0.000362985
PIN_scaffold_1_0759	9.473	54.11	2.577059127	0.002355326	4.77E-05
PIN_scaffold_7_1944	17.456	37.033	1.523389835	0.029549071	0.001251357
PIN_scaffold_6_1129	1.293	6.29	2.524533851	0.002232481	4.47E-05
PIN_scaffold_9_1243	5.156	16.74	1.985413075	0.02691992	0.001087185
PIN_scaffold_3_0185	6.24	17.716	1.909373211	0.000644759	1.01E-05
PIN_scaffold_7_0639	92.57	259.13	1.896872843	0.000366855	4.97E-06
PIN_scaffold_3_0178	17.166	56.45	2.257984948	0.000486584	7.03E-06
PIN_scaffold_5_1280	0.186	1.176	2.4092586	0.034859948	0.001558983
PIN_scaffold_4_0304	45.64	236.086	2.373617867	0.018552754	0.000663763
PIN_scaffold_7_1393	0.04	1.496	4.587714622	2.80E-05	2.21E-07

PIN_scaffold_1_0857	158.363	622.69	2.189755993	0.027501973	0.001119285
PIN_scaffold_10_0273	2.05	9.223	2.522548782	1.08E-07	3.98E-10
PIN_scaffold_7_2008	51.19	129.203	1.615763827	0.000545213	8.09E-06
PIN_scaffold_8_1369	75.586	167.3	1.618619293	0.039426419	0.001854056
PIN_scaffold_5_1600	24.183	76.72	1.897452594	0.014737865	0.00049792
PIN_scaffold_7_1474	178.696	493.626	1.823691869	0.002057446	4.04E-05
PIN_scaffold_13_1031	11.376	78.5	3.047111201	2.11E-05	1.57E-07
PIN_scaffold_3_0112	19.29	39.166	1.399448165	0.008629429	0.000242337
PIN_scaffold_8_0191	3.49	36.636	3.931650575	1.03E-24	1.21E-28
PIN_scaffold_6_0671	0.326	1.316	2.099650224	0.034853163	0.001555957
PIN_scaffold_13_1637	131.403	1063.43	3.348259092	8.98E-08	3.19E-10
PIN_scaffold_5_2249	0.883	3.92	2.29730917	0.039330322	0.001848001
PIN_scaffold_14_0978	0.276	3.286	3.097458587	0.013020427	0.000418029
PIN_scaffold_12_1430	1.596	4.783	1.876256923	0.043328896	0.002161114
PIN_scaffold_9_0545	197.003	443.216	1.561403537	0.00075548	1.23E-05
PIN_scaffold_5_0237	23.676	38.046	1.122872612	0.022506779	0.00085797
PIN_scaffold_3_0146	7.196	21.036	1.948826149	4.54E-06	2.66E-08
PIN_scaffold_10_0379	43.906	135.833	1.95783656	0.000799799	1.32E-05
PIN_scaffold_9_0046	10.413	25.713	1.724545162	0.000537723	7.94E-06
PIN_scaffold_11_0985	21.343	56.31	1.828782752	7.94E-06	5.12E-08
PIN_scaffold_12_1126	0.893	3.843	2.337322883	0.000673776	1.07E-05
PIN_scaffold_12_0372	22.696	153.656	2.719279604	0.002209776	4.42E-05

PIN_scaffold_10_2432	1.03	2.786	1.982615604	0.040680543	0.001955383
PIN_scaffold_5_1536	26.78	37.113	1.072772782	0.043285495	0.002155568
PIN_scaffold_10_0897	0.06	1.163	3.282166643	0.01502706	0.000512973
PIN_scaffold_10_2440	0.373	6.433	3.256349261	0.013773115	0.000450801
PIN_scaffold_10_2371	0.233	1.393	2.547962471	0.024344303	0.00094133
PIN_scaffold_11_1790	4.286	10.733	1.745317329	0.004424565	0.000102119
PIN_scaffold_7_1277	2.163	4.37	1.302862952	0.041960148	0.002059946
PIN_scaffold_2_0768	18.23	39.756	1.570088925	0.007156953	0.000189551
PIN_scaffold_5_1787	2.82	142.35	5.804054392	1.06E-23	1.65E-27
PIN_scaffold_11_1874	2.843	7.103	1.762465859	0.000372357	5.10E-06
PIN_scaffold_10_0293	12.903	28.356	1.535687034	0.002115171	4.18E-05
PIN_scaffold_13_1199	1.243	4.026	2.044346891	0.002967124	6.27E-05
PIN_scaffold_6_0365	5.963	1.303	-1.647715949	0.027875788	0.001147564
PIN_scaffold_7_1327	5.95	1.546	-1.423342967	0.012521665	0.000394191
PIN_scaffold_14_0745	37.173	9.786	-1.50140719	0.012113265	0.00037246
PIN_scaffold_11_1080	0.933	0.086	-2.553230272	0.00487542	0.000115397
PIN_scaffold_1_0986	2.29	0.153	-2.831193521	0.010458891	0.000307194
PIN_scaffold_9_1804	95.87	21.773	-1.575580489	0.004713952	0.000111023
PIN_scaffold_10_0717	4.63	1.226	-1.572942435	0.006760502	0.000175858
PIN_scaffold_8_0743	8.853	0.736	-2.68194547	0.004690578	0.000109923
PIN_scaffold_10_0614	2.93	0.183	-2.987230205	0.002460091	5.03E-05
PIN_scaffold_5_2698	146.236	33.923	-1.615607434	0.013431285	0.000435416

PIN_scaffold_10_1333	1.34	0.21	-2.085876552	0.002104127	4.15E-05
PIN_scaffold_8_2128	3.726	0.126	-3.53705622	0.001896979	3.64E-05
PIN_scaffold_5_2468	10.003	2.8	-1.281295537	0.025029065	0.000986071
PIN_scaffold_7_0998	23.466	4.07	-2.106207463	0.00021044	2.41E-06
PIN_scaffold_2_0598	10.94	0.183	-4.950723187	3.20E-13	4.51E-16
PIN_scaffold_14_0162	61.976	9.623	-2.116452392	0.025029065	0.000985174
PIN_scaffold_1_0544	1.65	0.02	-4.325961537	0.000429324	6.00E-06
PIN_scaffold_5_0073	6.373	1.343	-1.685649344	0.021392602	0.000802964
PIN_scaffold_7_1008	2.23	0.206	-2.755175493	0.000166944	1.86E-06
PIN_scaffold_10_2971	18	4.736	-1.41223039	0.002322808	4.68E-05
PIN_scaffold_12_1551	9.44	2.226	-1.613195281	0.039161138	0.001836985
PIN_scaffold_9_1317	7.17	1.71	-1.519203915	0.036322128	0.001648994
PIN_scaffold_8_1547	5.36	1.276	-1.638557129	0.028912815	0.001209453
PIN_scaffold_6_0581	4.723	1.063	-1.498857529	0.04244988	0.002094059
PIN_scaffold_6_0450	3.053	0.686	-1.622940954	0.030342161	0.001296501
PIN_scaffold_7_2564	2.19	0.343	-2.081274545	0.004124127	9.23E-05
PIN_scaffold_3_0784	4.28	0.333	-2.812466691	0.000467213	6.72E-06
PIN_scaffold_11_1654	1.913	0.333	-1.983224793	0.013921544	0.000457835
PIN_scaffold_13_1144	16.073	4.873	-1.26928135	0.035109902	0.001572904
PIN_scaffold_7_2381	1.196	0.2	-1.931854511	0.029187679	0.001224948
PIN_scaffold_6_0205	2.616	0.493	-1.603358081	0.026816143	0.001081443
PIN_scaffold_5_2990	12.46	3.346	-1.372523145	0.024986701	0.000981784

PIN_scaffold_9_1938	19.14	3.87	-1.702988186	0.042888564	0.002129101
PIN_scaffold_12_0084	72.023	23.576	-1.119393379	0.046416071	0.002364042
PIN_scaffold_7_1037	27.973	7.32	-1.406065673	0.041116874	0.001988153
PIN_scaffold_5_3230	5.72	1.11	-1.961887175	0.004983233	0.000118294
PIN_scaffold_2_0790	11.243	0.936	-2.934035686	1.04E-05	6.77E-08
PIN_scaffold_7_0097	15.48	5.32	-1.102557374	0.046416071	0.002363913
PIN_scaffold_8_0433	16.243	3.336	-1.709555024	0.005870688	0.000144916
PIN_scaffold_11_0865	61.056	10.293	-1.580747156	0.002171117	4.31E-05
PIN_scaffold_6_0789	46.1	9.736	-1.69973057	0.01602367	0.00055513
PIN_scaffold_8_1090	3.34	0.73	-1.69194657	0.000510825	7.50E-06
PIN_scaffold_10_0337	4.54	0.66	-2.159794209	0.037847951	0.001753479
PIN_scaffold_8_1423	2.22	0.393	-1.936990787	0.000530483	7.81E-06
PIN_scaffold_2_0526	2.843	0.366	-2.245244459	0.000234046	2.75E-06
PIN_scaffold_7_1676	40.273	10.816	-1.379198997	0.001499381	2.78E-05
PIN_scaffold_8_1609	21.296	3.383	-2.032694871	7.30E-06	4.59E-08
PIN_scaffold_11_0172	75.74	12.45	-2.071772578	0.002187823	4.35E-05
PIN_scaffold_7_2007	3.033	0.573	-1.838178272	0.00487542	0.00011532
PIN_scaffold_12_0844	16.996	5.706	-1.091340892	0.041330987	0.002006578
PIN_scaffold_10_0310	7.56	1.193	-2.113271407	2.38E-05	1.84E-07
PIN_scaffold_12_1513	6.356	0.733	-2.076226667	0.039583144	0.001867611
PIN_scaffold_10_0180	4.42	1.266	-1.278451061	0.047960653	0.002470808
PIN_scaffold_8_0222	16.15	1.12	-3.117887157	4.25E-05	3.73E-07

PIN_scaffold_9_0272	20.76	3.553	-1.690222579	8.84E-05	8.74E-07
PIN_scaffold_8_0227	0.54	0.023	-3.824984124	0.000149963	1.65E-06
PIN_scaffold_8_1039	7.4	1.92	-1.406704578	0.012574409	0.000396342
PIN_scaffold_7_0190	101.34	26.71	-1.515769793	0.000703102	1.13E-05
PIN_scaffold_5_2189	30.573	8.63	-1.359536495	0.02527646	0.001004907
PIN_scaffold_9_0972	8.526	1.563	-1.714150119	0.026604633	0.001067014
PIN_scaffold_13_0536	8.606	2.12	-1.498781994	0.027997792	0.001160241
PIN_scaffold_1_1171	22.913	3.73	-1.98089641	5.13E-06	3.07E-08
PIN_scaffold_8_2045	30.613	8.8	-1.278402981	0.01299164	0.000415075
PIN_scaffold_13_1147	3.806	0.89	-1.663978344	0.039159776	0.00183234
PIN_scaffold_8_1997	110.266	13.81	-2.434176316	0.000233886	2.74E-06
PIN_scaffold_13_0366	4.953	1.396	-1.291991049	0.024742571	0.000969293
PIN_scaffold_7_0920	326.47	80.943	-1.461569341	0.008065289	0.000220824
PIN_scaffold_5_2550	29.103	7.61	-1.344267242	0.004124127	9.25E-05
PIN_scaffold_11_0961	85.313	20.776	-1.550427702	0.019767494	0.00071726
PIN_scaffold_5_2626	35.016	8.676	-1.518071745	0.035733488	0.001609214
PIN_scaffold_11_1440	6.966	0.936	-2.504287281	4.45E-05	4.02E-07
PIN_scaffold_13_0854	9.33	1.423	-1.941327696	0.020045339	0.00072969
PIN_scaffold_13_1259	7.75	1.87	-1.521783923	0.009755347	0.000281957
PIN_scaffold_5_2495	1.91	0.48	-1.677037871	0.024637085	0.000960349
PIN_scaffold_13_0464	6.906	1.376	-1.863059915	0.003045666	6.45E-05
PIN_scaffold_5_2519	303.89	72.856	-1.576064039	0.001499381	2.79E-05

PIN_scaffold_9_0392	6.19	1.416	-1.662709832	0.012965564	0.000413735
PIN_scaffold_10_1220	100.513	24.183	-1.633589342	0.000184609	2.10E-06
PIN_scaffold_11_0203	4.246	0.716	-1.992232445	0.000982615	1.67E-05
PIN_scaffold_5_0719	7.403	1.01	-2.223874303	0.010674792	0.000316454
PIN_scaffold_7_0536	3.91	0.603	-2.200047261	0.000127268	1.35E-06
PIN_scaffold_3_0342	33.403	9.82	-1.251895609	0.008435613	0.000233945
PIN_scaffold_7_1489	6.406	0.98	-2.052037276	0.011886295	0.000359797
PIN_scaffold_7_2003	152.253	45.2	-1.282608536	0.048844741	0.002544971
PIN_scaffold_13_1623	19.07	3.83	-1.77509189	0.020081051	0.000731774
PIN_scaffold_4_1130	3.35	0.796	-1.5521523	0.018202893	0.000647691
PIN_scaffold_11_1609	14.12	2.986	-1.669085465	0.003185198	6.79E-05
PIN_scaffold_4_0993	10.583	3.736	-1.008889251	0.049145306	0.002570229
PIN_scaffold_7_1955	17.073	2.91	-2.01113285	0.001584947	2.98E-05
PIN_scaffold_9_1572	5.023	0.956	-1.706625474	0.049558407	0.002605383
PIN_scaffold_13_1033	1189.123	317.536	-1.457367597	0.042134761	0.002071735
PIN_scaffold_8_1594	5.29	1.503	-1.275491728	0.032219589	0.001400633
PIN_scaffold_10_1764	6.25	2.133	-1.063041475	0.041576522	0.002021924
PIN_scaffold_5_0563	1.82	0.223	-2.166364328	0.009841111	0.000285205
PIN_scaffold_3_0986	24.976	7.54	-1.097877575	0.035368819	0.001588647
PIN_scaffold_11_1347	5.05	1.113	-1.633832731	0.027634605	0.001133318
PIN_scaffold_7_1779	16.476	3.12	-2.091644009	0.008949633	0.000254475
PIN_scaffold_14_0590	9.103	2.093	-1.54061855	0.020922542	0.000778783

PIN_scaffold_7_1386	10.17	2.54	-1.407423346	0.005849788	0.000143943
PIN_scaffold_9_1436	11.89	1.366	-2.258289489	0.044265113	0.002218183
PIN_scaffold_10_1689	66.213	13.273	-1.770972563	0.000262507	3.19E-06
PIN_scaffold_9_2016	7.536	1.686	-1.598937556	0.02231431	0.000848895
PIN_scaffold_5_2317	34.816	8.36	-1.54554681	0.048421561	0.002507792
PIN_scaffold_8_1418	7.736	1.873	-1.580734418	0.027710229	0.001139666
PIN_scaffold_6_1015	16.89	6.016	-1.132621462	0.046974885	0.002399842
PIN_scaffold_10_0347	53.83	15.823	-1.273828193	0.000966428	1.63E-05
PIN_scaffold_7_2100	6.496	0.303	-3.412710088	0.000290573	3.68E-06
PIN_scaffold_7_1936	70.943	11.906	-2.034777272	0.000114713	1.16E-06
PIN_scaffold_12_0085	100.983	22.366	-1.683393374	0.007611939	0.000205736
PIN_scaffold_5_2020	0.596	0.053	-2.722413538	0.035273978	0.001581632
PIN_scaffold_7_1374	7.82	2.37	-1.2909573	0.039007842	0.001822184
PIN_scaffold_11_1816	8.446	2.146	-1.484160853	0.026816143	0.001081001
PIN_scaffold_10_0306	80.726	21.383	-1.464269909	0.048638948	0.002530449
PIN_scaffold_6_0542	68.113	23.296	-1.02853065	0.038097796	0.001767769
PIN_scaffold_10_2524	4.856	1.223	-1.521561674	0.029848289	0.001269569
PIN_scaffold_12_1387	5.516	1.02	-1.810711463	0.038682958	0.001800964
PIN_scaffold_13_0362	18.18	2.173	-2.539475879	0.000367724	5.01E-06
PIN_scaffold_1_0580	2.903	0.513	-1.95427399	0.001764906	3.34E-05
PIN_scaffold_5_0239	15.093	2.156	-2.182001597	0.000506176	7.37E-06
PIN_scaffold_1_1154	4.85	0.55	-2.462542313	0.001812381	3.46E-05

PIN_scaffold_5_1716	6.43	2.11	-1.219343586	0.036171261	0.001635993
PIN_scaffold_9_1358	6.72	1.29	-1.92396818	0.006734969	0.000173601
PIN_scaffold_10_1549	10.953	3.113	-1.383323813	0.027598548	0.001129569
PIN_scaffold_9_1131	1.563	0.146	-2.661896721	0.002902478	6.09E-05
PIN_scaffold_12_1710	100.883	20.036	-2.066883385	1.94E-09	5.31E-12
PIN_scaffold_12_0470	42.72	5.96	-2.289003696	3.53E-05	2.98E-07
PIN_scaffold_6_1523	4.823	1.166	-1.521421767	0.009634257	0.000277329
PIN_scaffold_7_0171	0.85	0.113	-2.212076396	0.003663681	8.03E-05
PIN_scaffold_7_0043	39.366	13.196	-1.163315243	0.020782142	0.000763817
PIN_scaffold_13_0999	8.15	2.843	-1.086094939	0.038387954	0.001784231
PIN_scaffold_7_1192	10.506	1.073	-2.51835696	0.007389809	0.000197423
PIN_scaffold_9_1414	12.633	1.523	-2.478740703	3.78E-05	3.22E-07
PIN_scaffold_7_0501	4.26	0.653	-2.033593723	0.042134761	0.002073577
PIN_scaffold_11_1163	2.113	0.3	-2.22848215	0.000302358	3.86E-06
PIN_scaffold_9_1094	4.77	1.19	-1.49888524	0.041666739	0.002029388
PIN_scaffold_9_0349	173.996	49.036	-1.492533177	0.00773719	0.000211237
PIN_scaffold_10_0333	3.583	0.823	-1.562822525	0.007474837	0.000201152
PIN_scaffold_9_0273	8.463	2.233	-1.447050651	0.013020427	0.000417571
PIN_scaffold_8_1647	6.803	1.683	-1.519811468	0.009721944	0.000280233
PIN_scaffold_14_0204	3.54	0.47	-2.234370194	0.002745082	5.71E-05
PIN_scaffold_6_0556	2.903	0.56	-1.725879688	0.030858372	0.001330611
PIN_scaffold_1_1082	13.066	2.253	-1.653878703	0.007629947	0.000207802

PIN_scaffold_10_0738	22.416	7.54	-1.129677819	0.033254021	0.001458589
PIN_scaffold_7_2287	2.583	0.26	-2.438159875	0.031438106	0.001364043
PIN_scaffold_12_0720	39.37	9.36	-1.906992409	0.000142523	1.54E-06
PIN_scaffold_7_0135	10.626	1.406	-2.290946518	0.000262507	3.19E-06
PIN_scaffold_11_0271	5.363	0.8	-2.173975235	0.000825097	1.36E-05
PIN_scaffold_3_1103	16.296	2.856	-2.088122379	7.24E-05	6.90E-07
PIN_scaffold_9_1083	12.53	2.383	-1.801111073	0.029549071	0.001251849
PIN_scaffold_14_0017	80.83	1.83	-4.740174434	3.85E-16	3.31E-19
PIN_scaffold_2_0926	6.616	1.136	-1.838736176	0.005215867	0.000125492
PIN_scaffold_8_0046	2.593	0.686	-1.373521938	0.037847951	0.001754697
PIN_scaffold_12_0962	6.936	1.546	-1.607853113	0.029753551	0.001264378
PIN_scaffold_10_0597	252.266	69.84	-1.228729675	0.046923626	0.002393558
PIN_scaffold_12_0718	23.726	6.236	-1.323124093	0.004424565	0.000102178
PIN_scaffold_8_1789	15.19	3.436	-1.610062464	0.025394196	0.001011681
PIN_scaffold_5_1501	60.053	16.943	-1.351475875	0.038387954	0.001782973
PIN_scaffold_5_1393	5.666	1.64	-1.29187535	0.048572251	0.002519046
PIN_scaffold_10_2743	7.363	1.51	-1.795035095	0.001955629	3.79E-05
PIN_scaffold_10_2800	25.673	6.903	-1.388781438	0.029195128	0.001229245
PIN_scaffold_3_1302	77.45	17.61	-1.59391973	0.042979619	0.002136545
PIN_scaffold_11_0570	12.08	2.756	-1.586021691	0.006477183	0.000164946
PIN_scaffold_7_1314	3.48	0.553	-2.183310091	1.46E-05	1.00E-07
PIN_scaffold_9_0204	6.616	1.146	-1.951386989	0.01153051	0.000346775

PIN_scaffold_6_0774	2	0.336	-1.943295785	0.001170554	2.08E-05
PIN_scaffold_5_2744	6.48	1.55	-1.485862864	0.027570463	0.001124226
PIN_scaffold_11_0385	2.866	0.65	-1.535237855	0.02463128	0.000959154
PIN_scaffold_5_1250	11.956	1.703	-2.112742048	0.045624725	0.002298789
PIN_scaffold_5_1964	25.973	3.496	-2.370336588	1.09E-07	4.09E-10
PIN_scaffold_13_1223	4843.67	753.106	-2.126744978	8.24E-05	8.01E-07
PIN_scaffold_6_0606	123.873	39.073	-1.137938429	0.012039312	0.00036772
PIN_scaffold_10_0759	23.536	7.21	-1.168167259	0.045746973	0.002312096
PIN_scaffold_8_1758	59.733	12.066	-1.70305172	0.011554942	0.000347962
PIN_scaffold_14_0083	2.216	0.446	-1.802852091	0.004129341	9.27E-05
PIN_scaffold_14_0087	6.866	0.963	-2.314083688	0.005414803	0.000130701
PIN_scaffold_10_2369	0.906	0.146	-2.018230462	0.015722506	0.000541626
PIN_scaffold_14_0501	4.383	0.77	-1.927930865	0.036644745	0.001676014
PIN_scaffold_9_1482	56.98	17.75	-1.176601363	0.028381767	0.001182805
PIN_scaffold_3_0362	115.07	36.673	-1.124839931	0.020922542	0.000776026
PIN_scaffold_12_1425	9.51	1.623	-2.029608496	0.004351868	9.96E-05
PIN_scaffold_6_0959	5.82	1.506	-1.394259211	0.019556239	0.000706539
PIN_scaffold_13_0254	14.406	3.616	-1.396996929	0.002662466	5.52E-05
PIN_scaffold_11_1147	9.976	2.456	-1.51185551	0.013976879	0.000460746
PIN_scaffold_12_0116	34.01	5.176	-2.176795505	5.18E-05	4.75E-07
PIN_scaffold_8_1292	22.753	6.59	-1.29615169	0.013919083	0.00045721
PIN_scaffold_5_3338	40.966	2.72	-3.629160568	7.21E-12	1.30E-14

PIN_scaffold_10_0079	5.033	0.576	-2.324039965	0.027365329	0.001110623
PIN_scaffold_13_1598	11.8	3.383	-1.202197825	0.012114031	0.000373459
PIN_scaffold_7_1798	13.953	4.893	-1.062118828	0.026094542	0.001043659
PIN_scaffold_13_1012	35.386	5.633	-2.081901217	1.47E-05	1.02E-07
PIN_scaffold_6_0680	8.856	1.293	-2.228901535	0.049100892	0.002562153
PIN_scaffold_10_1223	5.716	0.836	-2.111150734	0.025249781	0.001000997
PIN_scaffold_8_0260	2.87	0.693	-1.524875033	0.027966525	0.001155979
PIN_scaffold_5_2021	146.406	21.773	-2.058999218	0.027365329	0.001111586
PIN_scaffold_3_0935	4.63	1.086	-1.50897458	0.019289795	0.000694653
PIN_scaffold_7_1737	26.28	6.63	-1.498844333	0.015723052	0.000542259
PIN_scaffold_13_1414	21.266	0.446	-4.489853581	4.54E-08	1.51E-10
PIN_scaffold_10_0305	16.39	2.546	-2.163647188	0.007429565	0.000199356
PIN_scaffold_7_1979	1.903	0.536	-1.571521536	0.037555319	0.001730863
PIN_scaffold_5_2403	171.7	57.17	-1.106639071	0.049676818	0.002614762
PIN_scaffold_10_2755	2.976	0.506	-1.94478364	0.013679543	0.000446136
PIN_scaffold_8_1719	6.4	2.08	-1.159443327	0.030829733	0.00132456
PIN_scaffold_13_1076	293.76	24.3	-3.013372565	1.15E-07	4.41E-10
PIN_scaffold_6_1421	6.83	2.24	-1.113800794	0.048300815	0.002495879
PIN_scaffold_5_2752	2.603	0.623	-1.479899096	0.024687655	0.000964249
PIN_scaffold_10_1421	2.443	0.443	-1.871838035	0.011313716	0.000339372
PIN_scaffold_7_2190	3.433	0.673	-1.797341656	0.000909678	1.51E-05
PIN_scaffold_8_1866	57.27	10.396	-1.825655748	0.013402055	0.000433694

PIN_scaffold_8_0583	4.213	1.236	-1.265456079	0.015007137	0.000510697
PIN_scaffold_10_3086	1.953	0.336	-1.90116861	0.028240028	0.001174692
PIN_scaffold_8_0665	44.873	14.43	-1.187005313	0.034169199	0.001509408
PIN_scaffold_10_2542	4.113	0.406	-2.493278807	5.10E-07	2.43E-09
PIN_scaffold_13_1130	113.603	11.393	-2.696147242	0.000285702	3.56E-06
PIN_scaffold_12_0430	1.53	0.126	-2.749813508	0.005584719	0.000136112
PIN_scaffold_9_1860	1.536	0.27	-1.881550776	0.016222318	0.00056518
PIN_scaffold_9_0426	91.78	29.216	-1.227918521	0.001153843	2.05E-05
PIN_scaffold_12_1057	5.16	0.8	-2.155488965	0.00026972	3.31E-06
PIN_scaffold_1_0433	4.156	0.52	-2.412764725	0.005641161	0.000137807
PIN_scaffold_4_1106	22.823	4.226	-1.94038057	0.000959917	1.61E-05
PIN_scaffold_10_2425	8.746	0.91	-2.610732272	0.000925286	1.55E-05
PIN_scaffold_8_0621	4.723	1.083	-1.553852621	0.040374198	0.001925434
PIN_scaffold_10_0878	5.036	1.703	-1.030202148	0.048572251	0.00251939
PIN_scaffold_8_0149	2077.7	135.62	-3.219002978	0.000125843	1.32E-06
PIN_scaffold_9_0853	14.82	3.186	-1.599394498	0.02463128	0.000958927
PIN_scaffold_11_0227	8.42	2.156	-1.39665476	0.038852646	0.001813417
PIN_scaffold_4_0735	4.77	0.623	-2.236343597	0.003176474	6.76E-05
PIN_scaffold_9_1848	8.643	2.786	-1.104033272	0.021238526	0.000794692
PIN_scaffold_3_0857	6.266	1.32	-1.815089451	0.003493774	7.57E-05
PIN_scaffold_12_0879	4.31	0.883	-1.691383662	0.047397159	0.002432526
PIN_scaffold_8_0399	60.566	17.256	-1.351215383	0.020698095	0.00075951

PIN_scaffold_10_2712	26.026	5.38	-1.722332458	0.006310931	0.000158987
PIN_scaffold_5_0045	7.783	2.173	-1.304561978	0.047494285	0.002443072
PIN_scaffold_10_2392	9.043	0.516	-2.902517637	0.036322128	0.00164991
PIN_scaffold_7_0944	15.65	2.263	-2.170504134	0.004183332	9.41E-05
PIN_scaffold_5_1973	0.41	0.02	-3.014468973	0.000545187	8.07E-06
PIN_scaffold_5_0429	8.253	1.633	-1.766673869	0.002613066	5.40E-05
PIN_scaffold_6_0145	45.663	12.916	-1.374598847	0.000366855	4.97E-06
PIN_scaffold_6_0907	15.57	1.976	-2.415063071	0.000464146	6.65E-06
PIN_scaffold_5_3319	5.5	1.606	-1.276109009	0.048638948	0.002527491
PIN_scaffold_9_0080	70.723	14.923	-1.61904245	9.95E-05	1.00E-06
PIN_scaffold_5_2727	5.666	1.54	-1.415302638	0.012610932	0.000398603
PIN_scaffold_8_1240	4.46	1.02	-1.537339987	0.028240028	0.001174653
PIN_scaffold_9_1966	24.71	7.003	-1.347558097	0.004087707	9.10E-05
PIN_scaffold_6_1588	56.356	7.896	-2.316346671	1.35E-07	5.50E-10
PIN_scaffold_7_1810	21.213	6.623	-1.19211684	0.043307199	0.00215834
PIN_scaffold_9_0921	10	2.27	-1.629872271	0.009319577	0.000266814
PIN_scaffold_8_1641	7.833	2.03	-1.480357667	0.001383866	2.51E-05
PIN_scaffold_10_0852	17.51	4.316	-1.640091379	0.006590817	0.000168612
PIN_scaffold_8_1752	2.033	0.37	-1.821199932	0.016323276	0.00057061
PIN_scaffold_10_0313	5.233	0.856	-2.017968708	0.001105177	1.95E-05
PIN_scaffold_3_1192	2.49	0.103	-3.377246445	0.006221025	0.000156236
PIN_scaffold_10_0419	45.693	10.38	-1.764297651	0.000117258	1.20E-06

PIN_scaffold_12_0552	2.49	0.21	-2.54915949	0.02527646	0.001005016
PIN_scaffold_10_0719	8.956	1.523	-1.948206109	0.013158293	0.000424511
PIN_scaffold_10_1002	6.64	1.866	-1.363657952	0.015261467	0.000522763
PIN_scaffold_5_2416	7.89	1.746	-1.670177381	0.00071994	1.16E-05
PIN_scaffold_9_0521	23.27	6.91	-1.279018788	0.002136832	4.23E-05
PIN_scaffold_7_1214	5.816	1.146	-1.592249876	0.000583542	8.82E-06
PIN_scaffold_3_0918	6.513	1.82	-1.400521313	0.02084415	0.000768538
PIN_scaffold_5_0612	2.62	0.28	-2.538985632	0.00148923	2.76E-05
PIN_scaffold_7_1023	27.91	5.49	-1.679113053	0.040134349	0.001909293
PIN_scaffold_7_0384	7.11	1.853	-1.434425432	0.001930606	3.73E-05
PIN_scaffold_7_0267	23.86	5.943	-1.360127291	0.039161138	0.001836993
PIN_scaffold_8_0742	17.23	2.253	-1.874978769	5.33E-05	4.94E-07
PIN_scaffold_8_1893	1.816	0.433	-1.483404639	0.024344303	0.000940868
PIN_scaffold_3_0440	7.633	1.29	-2.177727038	0.000366855	4.99E-06
PIN_scaffold_13_0901	3.476	0.713	-1.906422038	0.007253496	0.000192932
PIN_scaffold_11_0281	3.923	1.056	-1.32882197	0.014355002	0.000477696
PIN_scaffold_10_2169	17.83	3.506	-1.754435935	0.005228174	0.000125992
PIN_scaffold_9_0094	9.523	1.79	-1.676178708	0.006749348	0.000174924
PIN_scaffold_9_1783	35.45	12.09	-1.032378921	0.031163687	0.001346211
PIN_scaffold_9_0948	2.426	0.29	-2.272542894	0.036217309	0.00163949
PIN_scaffold_9_1896	13.01	4.79	-1.308767465	0.01705273	0.000598774
PIN_scaffold_12_0002	25.416	4.023	-1.95455919	7.61E-05	7.37E-07

PIN_scaffold_1_0913	10.273	2.413	-1.540935238	0.013402055	0.000433945
PIN_scaffold_4_0395	26.816	4.666	-1.961052539	0.003743686	8.22E-05
PIN_scaffold_1_0301	7.876	1.276	-1.692183074	0.018525105	0.00066205
PIN_scaffold_7_0226	2.576	0.643	-1.401025911	0.040205593	0.001914253
PIN_scaffold_5_0717	2.316	0.456	-1.621192679	0.018439712	0.000657558
PIN_scaffold_5_2172	30.1	6.76	-1.679281494	0.004757696	0.000112239
PIN_scaffold_1_0734	2.55	0.216	-2.55024721	0.000604463	9.18E-06
PIN_scaffold_13_0943	6.946	1.103	-1.894156688	0.00075548	1.23E-05
PIN_scaffold_6_0584	93.386	14.1	-1.992895987	1.80E-05	1.31E-07
PIN_scaffold_5_2785	25.516	6.093	-1.42397156	0.02084415	0.000768104
PIN_scaffold_11_0954	13.023	3.866	-1.265705545	0.009970516	0.000289734
PIN_scaffold_5_1233	4.343	0.916	-1.695689982	0.030507964	0.001305969
PIN_scaffold_7_2559	11.09	2.69	-1.330387573	0.009437354	0.000270924
PIN_scaffold_9_1051	1.916	0.18	-2.53944271	0.010679128	0.000316999
PIN_scaffold_6_0632	3.18	0.383	-2.391979636	0.007330906	0.000195563
PIN_scaffold_10_1219	9.51	1.433	-1.948022532	0.049467554	0.00259481
PIN_scaffold_7_0600	4.876	0.516	-2.458420352	0.027638651	0.001134563
PIN_scaffold_7_0702	61.233	2.83	-3.636811854	8.95E-07	4.44E-09
PIN_scaffold_5_2866	10.216	2.316	-1.611267486	0.032285384	0.001404754
PIN_scaffold_8_1417	74.25	16.123	-1.719576458	0.000379559	5.23E-06
PIN_scaffold_10_0449	5.78	1.17	-1.776563818	0.005458925	0.000132406
PIN_scaffold_7_0034	13.36	3.173	-1.56064997	0.017128323	0.000602097

PIN_scaffold_7_1815	6.663	0.953	-2.194606935	0.001403135	2.56E-05
PIN_scaffold_11_1772	7.073	1.403	-1.770943005	0.012219421	0.000379831
PIN_scaffold_12_1146	4.463	0.803	-1.90670573	0.008896627	0.000252621
PIN_scaffold_11_0759	12.32	1.636	-2.063078321	4.04E-05	3.52E-07
PIN_scaffold_13_0847	36.833	2.27	-3.363497447	1.56E-05	1.10E-07
PIN_scaffold_9_0355	49.52	9.983	-1.802250328	0.001058452	1.84E-05

Supplementary Table.4 Statistics of target gene prediction for 30 differentially expressed miRNAs

Type	miRNA count	Target count	Targeting relationships count
CDS	30	12211	13125
3'UTR	30	2254	4412
